# Distribution station inspection robot with modular manipulator

**DOI:** 10.1016/j.ohx.2025.e00652

**Published:** 2025-04-24

**Authors:** Qihui Yu, Qinglong Zhang, Guoxin Sun, Ripeng Qin

**Affiliations:** College of Mechanical Engineering, Inner Mongolia University of Science and Technology, Baotou 014010, China

**Keywords:** Robotics, Modularization, Power distribution substation, Open-science, Low-cost

## Abstract

Substation inspection robots play a vital role in ensuring the safety of power grid systems and improving operational efficiency. However, existing inspection robots often face challenges such as limited flexibility at the mechanical end-effector, slow tool-switching speeds, and an inability to meet diverse operational demands, which restrict their efficiency and adaptability. To overcome these limitations, this paper introduces a substation inspection robot equipped with a modular robotic arm. The robot incorporates a coaxially aligned end-effector design, enabling rapid tool switching across three operational modes via a single-degree-of-freedom drive. This design allows for precise operation of control devices, including push buttons, rotary switches, and draw-out circuit breakers, significantly enhancing both operational flexibility and efficiency. Moreover, the robot adheres to the principles of low cost, open-source accessibility, and modularity, making it particularly suitable for indoor substation inspection tasks and operational validations. Additionally, it provides a versatile hardware platform for the development of algorithms and related research. Experimental results confirm the robot’s operational performance across a variety of scenarios, offering solid technological support for the realization of fully unmanned substation operations.

## Graphical abstract

**Figure:**
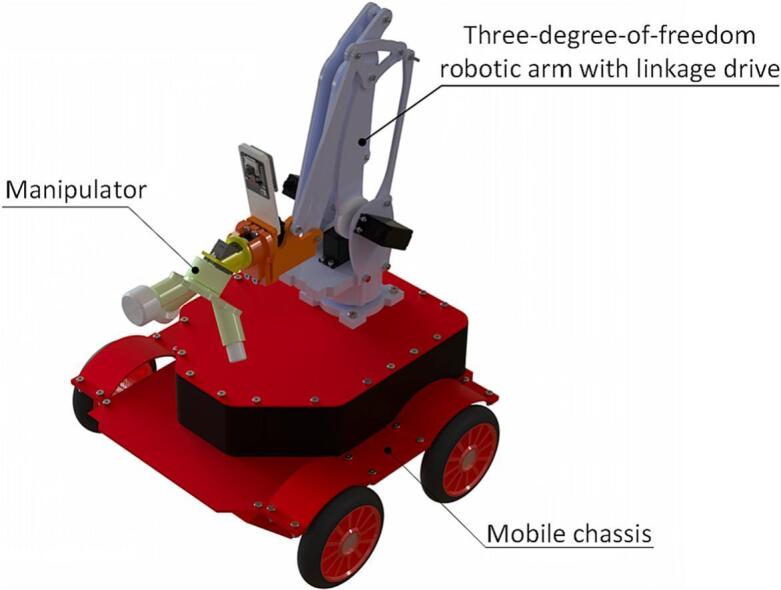

## 1 Specifications table

**Table:** 

Hardware name	*The name of the hardware that you have invented/customized*
Subject area	• Engineering and materials science
Hardware type	• Mechanical engineering and materials science
Closest commercial analog	Distribution cabinet operating robots
Open source license	GNU General Public License v3.0
Cost of hardware	2000￥
Source file repository	https://doi.org/10.17605/OSF.IO/TPGE9

## 2 Hardware in context

The inspection of substation equipment constitutes a critical component of routine maintenance in power systems, playing a significant role in ensuring power supply safety, improving operational efficiency, and reducing maintenance costs [1]. Such inspections enable the timely detection of equipment defects and potential safety hazards, thereby preventing power outages caused by equipment failures [2]. However, most substations still rely on manual inspection methods, which are notable for their high labor intensity, low efficiency, and elevated rate of misjudgment [3]. Moreover, the substation environment may contain colorless, odorless, and toxic gas leaks, posing severe threats to the safety of inspection personnel [4]. As a result, the development of inspection robots to replace manual operations has emerged as a pivotal solution to these challenges.

Substation inspection robots are intelligent, autonomous mobile devices designed to perform equipment status monitoring (e.g., meter reading recognition) and equipment operations (e.g., push buttons, rotary switches, and draw-out circuit breakers) in high-risk or repetitive power inspection tasks [5,6]. Currently, inspection robots are primarily categorized into four types: rail-mounted, wheeled, legged, and tracked robots. Rail-mounted robots are well-suited for standardized inspections along fixed routes and high-precision detection scenarios; however, they require fixed tracks, incur high deployment costs, and are limited to inspection tasks without the capability for live-line operations [7]. Wheeled robots are ideal for flat indoor environments, offering advantages such as low power consumption and long endurance; however, they exhibit limited adaptability to complex terrains. Legged robots excel in unstructured terrains but are characterized by high energy consumption, complex structures, and difficulties in achieving low-cost deployment [8]. Similarly, tracked robots are also suitable for unstructured terrains, but their flexibility and efficiency remain areas for improvement.

**Fig. 1:**
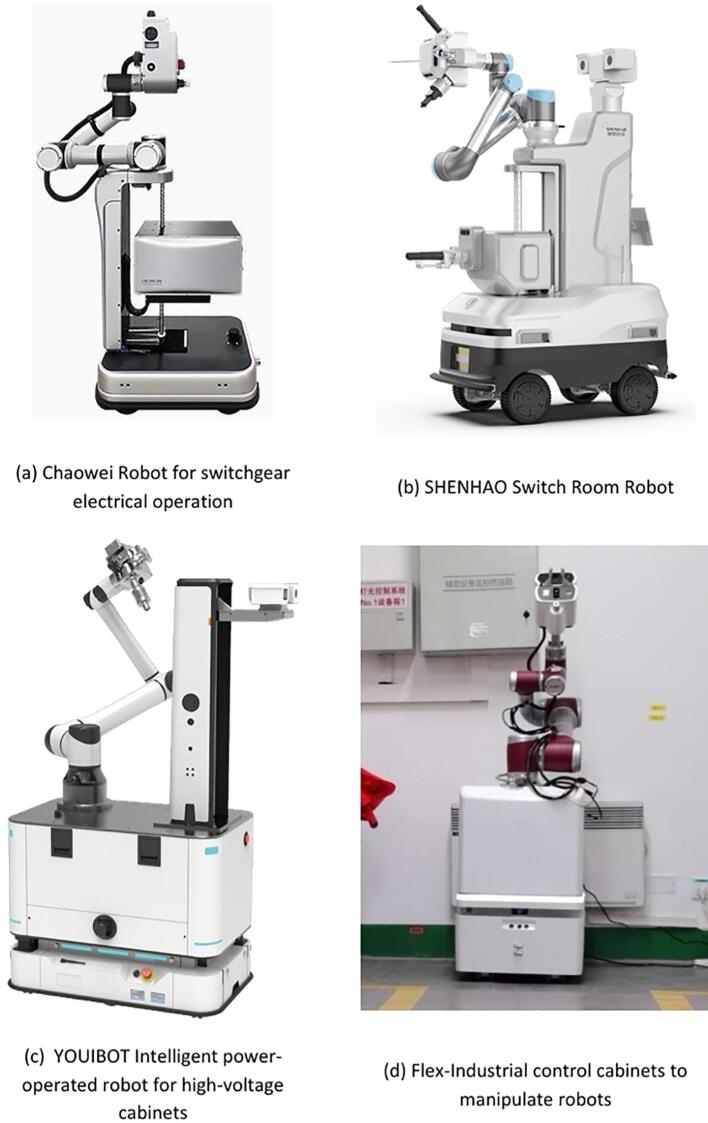
Existing inspection robots.

In recent years, significant progress has been made by research institutions and companies worldwide in the field of substation inspection robots. For example, the Shandong Electric Power Research Institute (SEPRI) began researching substation inspection robots as early as 1999. The initial prototype was capable of detecting equipment thermal defects, monitoring switchgear status, and performing automatic meter reading. However, it was constrained by fixed-track navigation and high costs [9]. The live-switch operation robot developed by SuperDroid Technology, as shown in Fig. 1(a), is primarily applied in high-voltage substation scenarios and can perform remote-controlled switching operations. However, its end-effector is relatively heavy, resulting in limited operational flexibility [10]. Robots developed by Shenhao Company [11] and Youai Zhihui Company [12], as shown in Fig. 1(b) and (c), have made improvements in inspection and operational functionality, but their end-tool switching speed remains slow, limiting their ability to efficiently meet diverse operational requirements. The industrial control cabinet operation robot launched by Huanmeng Technology, as shown in Fig. 1(d), can automatically perform cabinet door and button operations. However, since its modular tools are stored on the chassis, the robotic arm is required to move to the tool area on the chassis for replacement, which reduces operational efficiency [13].

Despite the functional and performance advancements of existing inspection robots, several shortcomings remain. The end tools of robotic arms in current inspection robots are usually fixed, making rapid switching difficult and leading to low operational efficiency, which hinders their ability to handle diverse substation equipment operations. Some robots adopt modular tool designs, but the tools are stored on the chassis, requiring the robotic arm to move to the switching area for replacement, thus increasing operation time and reducing overall efficiency. Many existing robots are designed for specific scenarios, hindering efficient operation in complex or dynamic environments. Additionally, some robots have complex designs, high manufacturing costs, and are difficult to maintain, restricting their widespread application and adoption.

To address the aforementioned issues, this paper proposes a substation inspection robot based on a modular robotic arm. The robot utilizes a wheeled mobile platform, making it suitable for indoor substation environments and capable of efficiently performing a variety of operational tasks, including push-button switches, rotary switches, and draw-out circuit breakers. Its main innovations are summarized as follows: it is equipped with a modular robotic arm featuring a coaxially aligned design, allowing rapid switching between three end-effectors through a single-degree-of-freedom drive, which significantly enhances operational flexibility and efficiency. The robot adheres to principles of low cost, open-source accessibility, and modular design, which reduces manufacturing and maintenance costs while offering a versatile hardware testbed for algorithm development and related studies. Additionally, the modular design enables the robot to adapt to diverse substation equipment operation requirements, supports functional upgrades and tool replacement, and provides a new technological pathway for achieving unmanned substation inspections.

Compared with traditional inspection robots, the proposed modular robotic arm not only effectively addresses the challenge of slow end-tool switching but also meets the diverse operational demands of substation equipment. It significantly enhances inspection efficiency, reduces equipment manufacturing and maintenance costs, and ensures the safety of workers. This research offers practical and viable technical support for the intelligent operation and maintenance of future substations.

## 3 Hardware description

**Fig. 2:**
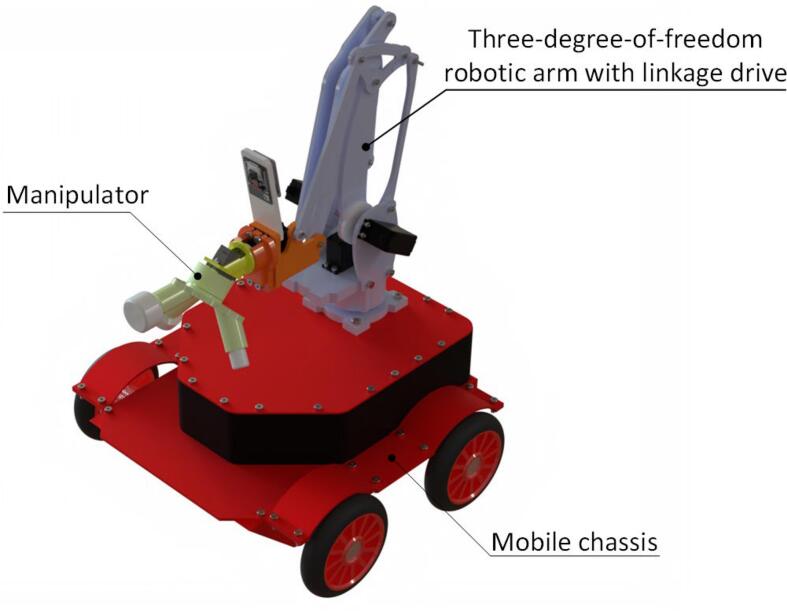
Overall view of the mobile robot.

**Fig. 3:**
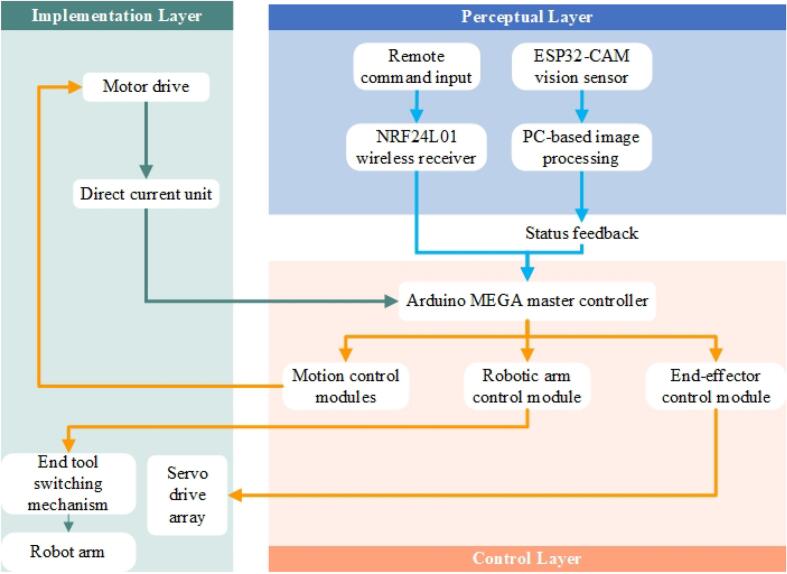
Block diagram of overall system architecture.

The substation inspection and operation robot is an intelligent system integrating perception, control, and actuation, whose hardware design demands a balance among functionality, reliability, and cost-effectiveness. As depicted in Fig. 2, the proposed inspection and operation robot follows the system framework shown in Fig. 3, which primarily consists of three layers: the perception layer, the control layer, and the actuation layer. These layers function collaboratively to perform inspection and operation tasks. A detailed explanation of the robot’s hardware architecture is presented in this section.

The perception layer forms the foundation of the robot’s hardware system, which is responsible for the collection and transmission of environmental information. In this study, the ESP32-CAM module is adopted as the primary visual perception device, owing to its low cost and high level of integration, and is capable of real-time video transmission and image processing. The robot transmits the captured image data to a PC, enabling remote monitoring and control by the operator. However, the ESP32-CAM module exhibits limited performance in low-light environments. To address this issue, a supplementary lighting system is implemented to improve image quality in low-light conditions. The lighting system is remotely controlled via the PC, ensuring operators can clearly observe the operation panels of distribution cabinets in such scenarios. Additionally, the perception layer can be further enhanced by integrating additional sensors, such as temperature and humidity sensors or gas detection sensors, to enhance environmental perception capabilities and offer more comprehensive data support for inspection tasks.

The control layer serves as the core of the robot’s hardware system, responsible for coordinating the functions of the perception layer and the actuation layer. In this study, the Arduino MEGA2560 is employed as the main control unit, integrated with the V2 expansion board to support a modular design, enabling the convenient connection of multiple peripheral modules. The control layer receives commands from a remote controller via a wireless communication module and converts them into specific control signals for the actuation layer. To improve scalability, the control layer includes multiple reserved interfaces for future expansion. Moreover, its software architecture is developed on an open-source platform, facilitating rapid algorithmic iteration and functionality extensions. Compared to traditional closed-loop control systems, this design reduces development costs while offering researchers a flexible platform for hardware experimentation.

The actuation layer is a critical subsystem of the robot responsible for executing practical tasks and comprises the mobile chassis, robotic arm, and modular end-effector. The mobile chassis employs a wheeled structure driven by 12 V DC motors, which features low power consumption and extended endurance, rendering it well-suited to the flat-floor environments typically found in indoor substations. The chassis design carefully accounts for weight distribution and hardware layout, with the upper layer allocated to the power supply and the lower layer reserved for mounting the main control board and other electronic components. This layered configuration not only optimizes the center of gravity but also improves hardware maintainability.

### 3.1 Mechanical part

The inspection and operation robot consists of three main components: a mobile chassis, a three-degree-of-freedom robotic arm actuated through linkage mechanisms, and an end-effector. The overall structure of the robot is constructed from aluminum alloy materials and enhanced with 3D printing technology.

**Fig. 4:**
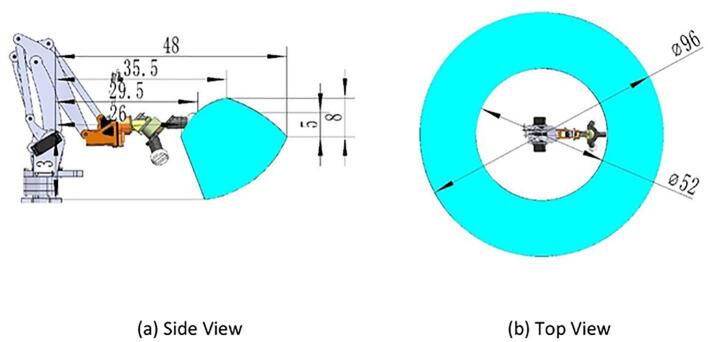
Illustration of robotic arm workspace.

The robotic arm features a three-degree-of-freedom design actuated through linkage mechanisms, with servos centrally mounted on the base. The parallelogram linkage mechanism enables precise control of the end-effector. Compared to traditional robotic arms, this design effectively minimizes the robotic arm’s inertia, thereby enhancing the stability and efficiency of its movements. The workspace of the robotic arm has been optimized (as shown in Fig. 4), allowing it to reach all operational zones of the distribution cabinet, ensuring the robot’s adaptability to a wide range of operation tasks.

The modular end-effector is a key innovation in the hardware design proposed in this study. The end-effector features a coaxial and collinear configuration, enabling rapid switching among three tools driven by a single degree of freedom. Its main body comprises a circular array structure with three hexagonal plug-in sockets designed to interface with different functional operating tools. This plug-in socket design minimizes rotational errors during tool switching, ensuring precise transitions. Magnets connect the main body to the operating tools, providing tool stability while facilitating quick replacement and expansion. Experimental results demonstrate that the end-effector efficiently performs tasks such as operating button switches, knob switches, and draw-out circuit breakers, thus significantly enhancing the robot’s adaptability and operational efficiency.

The end-effector combines a rotating disk with corresponding operational tools to implement a modular design, allowing it to adapt to switches of varying shapes on distribution cabinets. This approach overcomes the limitations of traditional end-effectors, which are typically limited to operating a single type of switch. Due to the structural complexity of the end-effector, 3D printing technology was employed to fabricate complex components with high efficiency, thereby reducing manufacturing difficulty and improving production efficiency.

To ensure the reliability and safety of the modular end-effector under working conditions, this study conducted a structural force analysis on the main structure and its operational components. Finite element analysis (FEA) was conducted to simulate the stress conditions of the end-effector across various operational scenarios. By analyzing the stress distribution and deformation of the end-effector, the analysis confirmed that the design meets the strength and durability requirements.

#### 3.1.1 Analysis method and material parameters

The material utilized for 3D printing is ABS, which has a flexural modulus of 1880 MPa and a flexural strength of 62 MPa. The analysis assumes that the end-effector operates under static working conditions while neglecting the influence of dynamic loads.

#### 3.1.2 Loading conditions and boundary conditions

**1. Button Switch Operation**

**Fig. 5:**
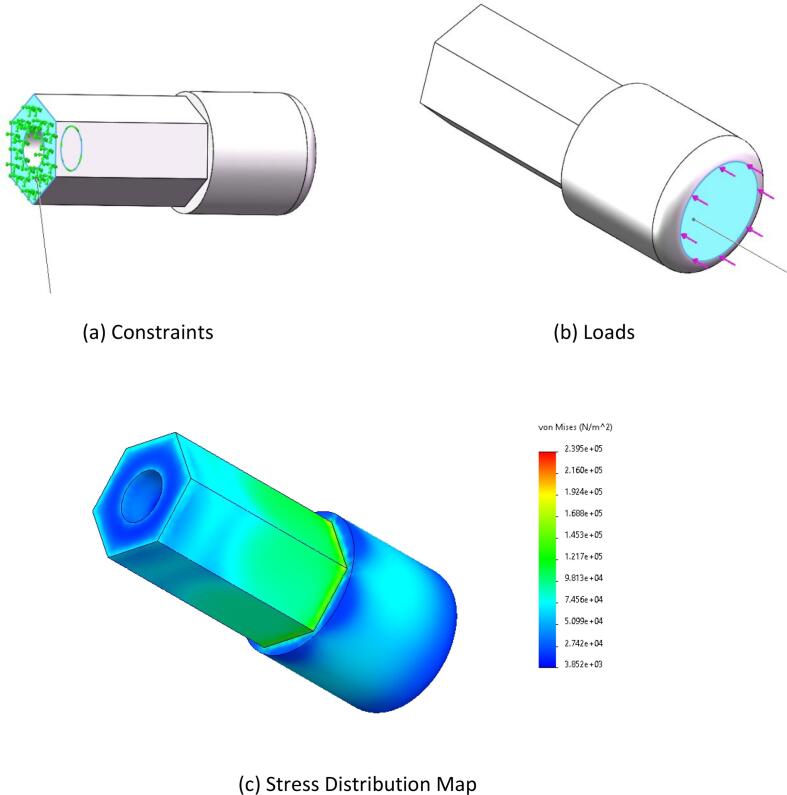
Pushbutton switch static stress.

The loading condition during the button-switch operation of the end-effector was analyzed. The required pressing force to operate the button switch is 8 N. With a safety factor of 1.5, the applied load was set to 12 N. The base of the hexagonal prism was defined as the fixed point (as shown in Fig. 5(a)), and a vertical force of 12 N was applied to the inner bottom surface of the hexagonal prism along the direction shown in Fig. 5(c).

**2. Knob Switch Operation**

**Fig. 6:**
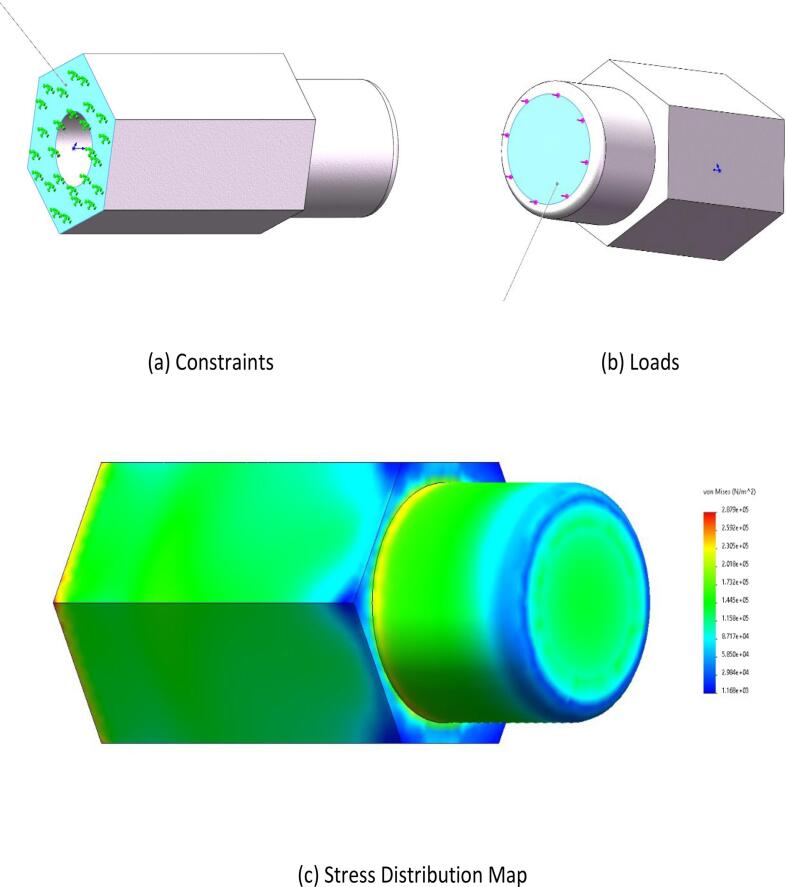
Operating instructions static stress.

The force condition during the knob-switch operation was simulated. Operating the knob switch requires a torque of 8 N·m. Considering a safety factor of 1.5, the applied torque was set to 12 N·m. The hexagonal prism was defined as the fixed point (as shown in Fig. 6(a)), and a 12 N·m torque was applied along the direction indicated in Fig. 6(b).

**3. Finger Manipulation Operation**

**Fig. 7:**
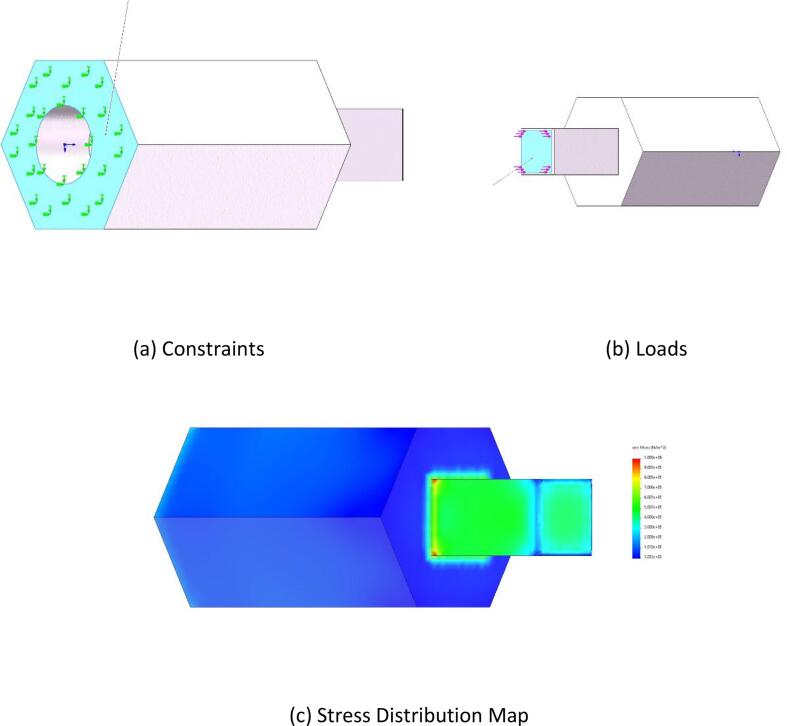
Square hole switch static stress.

The loading condition of the end-effector’s finger during operation was analyzed. The required pressing force for finger operation is 8 N. With a safety factor of 1.5, the applied load was set to 12 N. The base of the hexagonal prism was defined as the fixed point (as shown in Fig. 7(a)), and a vertical force of 12 N was applied to the inner bottom surface of the hexagonal prism along the direction specified in Fig. 7(c).

**4. Square-Hole Switch Operation**

**Fig. 8:**
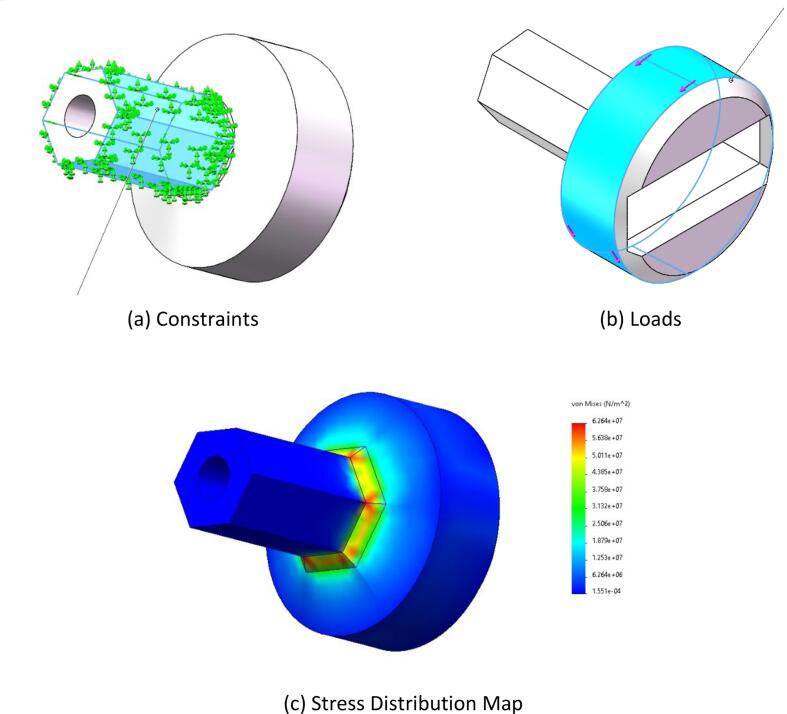
Rotary switch static stress.

The force condition during the rotation of a square-hole switch by the end-effector was simulated. The torque required to rotate the square-hole switch is 8 N·m. With a safety factor of 1.5, the applied torque was set to 12 N·m. The hexagonal prism was defined as the fixed point (as shown in Fig. 8(a)), and a 12 N·m torque was applied along the direction shown in Fig. 8(b).

**5. Force Analysis of the End-Effector Body**

**Fig. 9:**
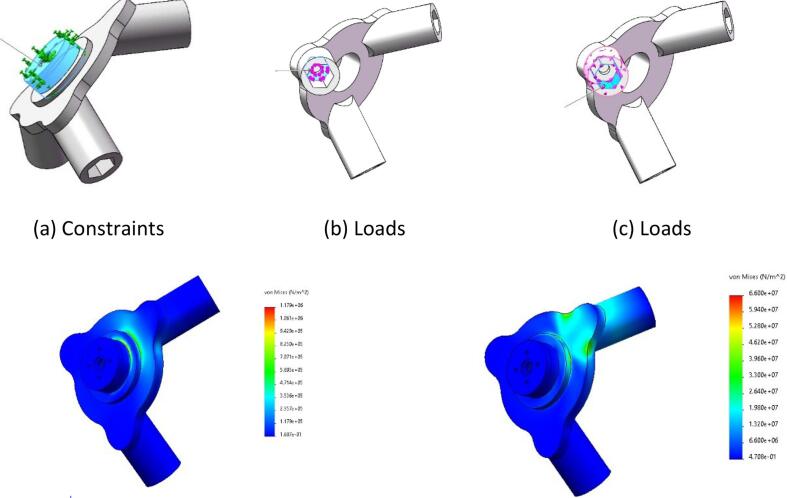
Manipulator body static stress.

A force analysis of the modular end-effector body was performed to simulate its stress condition during the operation of a draw-out circuit breaker. The end-effector body was defined as the fixed point (as shown in Fig. 9(a)), and loads were applied corresponding to the following operation scenarios:

• **Button Switch Operation:** A force of 12 N was applied along the direction shown in Fig. 9(b).
• **Knob Switch Operation:** A torque of 12 N·m was applied along the direction shown in Fig. 9(c).

#### 3.1.3 Analysis results

• **Button Switch Operation:** As shown in Fig. 5(c), the stress distribution is primarily concentrated in the middle of the component. The maximum stress observed is 0.24 MPa, which is well below the flexural strength of the material. This indicates that the design meets the strength requirements.
• **Knob Switch Operation:**Fig. 6(c) illustrates that the stress distribution is concentrated at the component’s connection point. The maximum stress recorded is 62.7 MPa, which remains within the flexural strength of the material, demonstrating the safety and reliability of the design.
• **Finger Manipulation Operation:** As presented in Fig. 7(c), the stress distribution is concentrated at the component’s connection point. The maximum stress observed is 0.29 MPa, which is below the flexural strength of the material, confirming that the design is both safe and reliable.
• **Square-Hole Switch Operation:** As shown in Fig. 8(c), the stress distribution is concentrated at the base of the hexagonal prism. The maximum stress is 1 MPa, which does not exceed the flexural strength of the material, verifying that the design is safe and reliable.
• **Force Analysis of the End-Effector Body:** Under the button-switch operation conditions, as shown in Fig. 9(d), the stress distribution is concentrated at the connection points of the components. The maximum stress is 1.18 MPa, which fulfills the design requirements. Similarly, under the knob-switch operation conditions, as shown in Fig. 9(e), the stress distribution is also concentrated at the connection points. The maximum stress measured is 46.2 MPa, which meets the required design specifications.

### 3.2 Electronic part

The electronic system employs the Arduino [14] platform, which offers open-source accessibility and a user-friendly programming environment, facilitating system development. The inspection robot is equipped with an Arduino MEGA2560 development board paired with a V2 expansion board, an ESP32-CAM development board, and an Arduino Pro Mini development board. Additionally, the robot incorporates four brushless DC motor controllers, four DC motors, and two NRF24L01+ wireless transceiver modules. The Arduino Pro Mini development board functions as the remote-control terminal, transmitting data to the robot via the NRF24L01+ wireless transceiver module. On the robot side, the Arduino MEGA2560 development board receives the data and controls the operations of the mobile chassis and the robotic arm. Simultaneously, the ESP32-CAM development board transmits real-time video, which is displayed on a PC to enhance inspection efficiency.

The inspection robot is powered by rechargeable lithium batteries, offering advantages such as low cost, environmental sustainability, and ease of maintenance. The Arduino MEGA2560 serves as the main control board, with the V2 expansion board enhancing system extensibility. The mobile chassis is driven by 12 V DC motors controlled by an L298N module, while the robotic arm is actuated by three servos. The end-effector is operated by two additional servos. Real-time video transmission is again performed by the ESP32-CAM, providing clear visual feedback to support inspection tasks.

## 4 Design files summary

The design files for the inspection robot used in power distribution stations are divided into two categories, optimized for aluminum alloy and 3D-printed materials. The mobile chassis is constructed from aluminum alloy, while the robotic arm and end-effector are fabricated using 3D-printed materials. To accommodate user preferences and diverse manufacturing capabilities, the design files are compatible with a wide range of materials. However, given the intricate design of the end-effector and the lightweight requirements of the robotic arm, the use of 3D-printed or other lightweight materials is recommended to simplify manufacturing and reduce costs. The design files are provided in.stl and.step formats, as detailed in Table 1.

**Table 1:** Design files to build the Inspection Robot.

Design file name	File type	Open source license	Location of the file
Bottom baffle	.step	GNU General Public License v3.0	https://osf.io/pfjsy
Bottom front fender	.step	GNU General Public License v3.0	https://osf.io/tje62
Bottom left baffle	.step	GNU General Public License v3.0	https://osf.io/tje62
Bottom rear fender	.step	GNU General Public License v3.0	https://osf.io/f48jv
Bottom right baffle	.step	GNU General Public License v3.0	https://osf.io/wydpa
Intermediate plate	.step	GNU General Public License v3.0	https://osf.io/4qnpy
Mudguard	.step	GNU General Public License v3.0	https://osf.io/6hck5
Top backsplash	.step	GNU General Public License v3.0	https://osf.io/fscny
Top baffle	.step	GNU General Public License v3.0	https://osf.io/5nuvc
Top front bezel	.step	GNU General Public License v3.0	https://osf.io/mc962
Top left and right baffles	.step	GNU General Public License v3.0	https://osf.io/7txmc
ActuatorⅠ	.stl.step	GNU General Public License v3.0	ActuatorⅠ
ActuatorⅡ	.stl.step	GNU General Public License v3.0	ActuatorⅡ
ActuatorⅢ	.stl.step	GNU General Public License v3.0	ActuatorⅢ
Base plate Ⅰ	.stl.step	GNU General Public License v3.0	Base plate Ⅰ
Base plate Ⅱ	.stl.step	GNU General Public License v3.0	Base plate Ⅱ
Base plate Ⅲ	.stl.step	GNU General Public License v3.0	Base plate Ⅲ
Base plate Ⅳ	.stl.step	GNU General Public License v3.0	Base plate Ⅳ
Base plate Ⅴ	.stl.step	GNU General Public License v3.0	Base plate Ⅴ
Base plate Ⅵ	.stl.step	GNU General Public License v3.0	Base plate Ⅵ
Base plate Ⅶ	.stl.step	GNU General Public License v3.0	Base plate Ⅶ
Big arm and small arm connection	.stl.step	GNU General Public License v3.0	Big arm and small arm connection
Big arm left side attachment plate Ⅰ	.stl.step	GNU General Public License v3.0	Big arm left side attachment plate Ⅰ
Big arm left side attachment plate Ⅱ	.stl.step	GNU General Public License v3.0	Big arm left side attachment plate Ⅱ
Big arm right side attachment plate Ⅰ	.stl.step	GNU General Public License v3.0	Big arm right side attachment plate Ⅰ
Big arm right side attachment plate Ⅱ	.stl.step	GNU General Public License v3.0	Big arm right side attachment plate Ⅱ
Camera bracket	.stl.step	GNU General Public License v3.0	Camera bracket
Left side limit plate	.stl.step	GNU General Public License v3.0	Left side limit plate
Left side support plate	.stl.step	GNU General Public License v3.0	Left side support plate
Manipulator body	.stl.step	GNU General Public License v3.0	Manipulator body
Right side limit plate	.stl.step	GNU General Public License v3.0	Right side limit plate
Right side support plate	.stl.step	GNU General Public License v3.0	Right side support plate
Rudder support	.stl.step	GNU General Public License v3.0	Rudder support
Servo connection	.stl.step	GNU General Public License v3.0	Servo connection
Servo connector	.stl.step	GNU General Public License v3.0	Servo connector
Small arm attachment plate Ⅰ	.stl.step	GNU General Public License v3.0	Small arm attachment plate Ⅰ
Small arm attachment plate Ⅱ	.stl.step	GNU General Public License v3.0	Small arm attachment plate Ⅱ

The design file of the distribution station inspection robot is divided into two different units, aluminum alloy material, and 3D printing material, in which the mobile chassis is machined from aluminum alloy material and the robotic arm and manipulator are machined from 3D printing material. Due to different user preferences and processing and manufacturing capabilities, the same design file can be processed with almost any material, but considering the complex design of the manipulator and the weight of the arm, it is recommended to use 3D printing materials or lightweight materials for processing to reduce the complexity of processing as well as the cost. In Table 1 the design files are provided in.stl and.step formats.

## 5 Bill of materials summary

To improve readability for users, the bill of materials (BOM) is divided into four sections: Table 2 presents the essential components for constructing the mobile chassis; Table 3 specifies the 3D-printed parts required for assembling the robotic arm and end-effector; Table 4 lists the basic electronic components necessary for building the circuit; and Table 5 details the mechanical elements, including screws and nuts.

**Table 2:** Bill of aluminum material.

Designator	Number	Cost per unit −currency	Total cost − currency	Source of materials	Material type
Top baffle	1	20	20	https://www.taobao.com	Metal
Top left and right baffles	2	15	30	https://www.taobao.com	Metal
Top front bezel	1	10	10	https://www.taobao.com	Metal
Top backsplash	1	5	5	https://www.taobao.com	Metal
Intermediate plate	1	30	30	https://www.taobao.com	Metal
Bottom front fender	1	10	10	https://www.taobao.com	Metal
Bottom left baffle	1	13	13	https://www.taobao.com	Metal
Bottom right baffle	1	13	13	https://www.taobao.com	Metal
Bottom rear fender	1	5	5	https://www.taobao.com	Metal
Bottom baffle	1	25	25	https://www.taobao.com	Metal
mudguard	4	5	20	https://www.taobao.com	Metal
Total			181

**Table 3:** Bill of 3D printing materials.

Description	Material	Amount [g]	Time [min]	Number
Base plate Ⅰ	PLA	21.65	36	1
Base plate Ⅱ	PLA	4.33	15	1
Base plate Ⅲ	PLA	1.97	10	1
Base plate Ⅳ	PLA	8.95	22	1
Base plate Ⅴ	PLA	8.25	23	1
Base plate Ⅵ	PLA	12.28	27	1
Base plate Ⅶ	PLA	30.16	48	1
Right side support plate	PLA	9.31	20	1
Left side support plate	PLA	10.7	22	1
Big arm right side attachment plate Ⅰ	PLA	14.57	24	1
Big arm right side attachment plate Ⅱ	PLA	6.65	16	1
Big arm right side attachment plate Ⅰ	PLA	4.38	14	1
Big arm right side attachment plate Ⅱ	PLA	15.89	26	1
Right side limit plate	PLA	9.82	19	1
Left side limit plate	PLA	5.42	14	1
Servo connection	PLA	1.09	15	1
Small arm attachment plate Ⅰ	PLA	6.58	17	1
Small arm attachment plate Ⅱ	PLA	40.39	56	2
Big arm and small arm connection	PLA	5.13	15	1
Rudder support	PLA	6.89	26	1
Servo connector	PLA	8.81	25	1
Manipulator body	PLA	35.74	78	1
ActuatorⅠ	PLA	7.43	24	1
ActuatorⅡ	PLA	10.17	30	1
ActuatorⅢ	PLA	3.5	20	1
Camera bracket	PLA	6.42	17	1
Total		296.48	659

**Table 4:** Bill of electronic materials.

Description	Number	Cost per unit(￥)	Total cost(￥)	Source of materials
Arduino MEGA 2560	1	295	295	https://www.taobao.com
MEGAV2 Expansion Board Veneer	1	63	63	https://www.taobao.com
L298N	2	6.8	13.6	https://www.taobao.com
Metal Pushbutton Switches	1	14.5	14.5	https://www.taobao.com
NRF24L01	2	5.5	11	https://www.taobao.com
Arduino pro mini	1	46.88	46.88	https://www.taobao.com
Toggle switch	1	0.12	0.12	https://www.taobao.com
3-pin, 2-position, single-connected pushbutton switch	2	8.92	17.84	https://www.taobao.com
Button	1	23	23	https://www.taobao.com
PS2 rocker	2	3.35	6.7	https://www.taobao.com
18,650 batteries	2	18.8	37.6	https://www.taobao.com
2 Series 18,650 Battery Pack	1	2.8	2.8	https://www.taobao.com
Skateboard wheels	4	7.9	31.6	https://www.taobao.com
MG996	4	13.5	54	https://www.taobao.com
SG90	1	10.6	10.6	https://www.taobao.com
Motor	4	262.4	1049.6	https://www.taobao.com
12VBattery 6000 mAh	1	78	78	https://www.taobao.com
AMS1117-3.3 Step-Down chip	1	0.41	0.41	https://www.taobao.com
Total			1756.25

**Table 5:** Bill of mechanic materials.

Designator	Component	Qty	Cost per unit(￥)	Total cost(￥)	Material
Hexagon socket head cap screws	M4 × 8 mm	50	5.2	5.2	steel
Hexagon socket head cap screws	M4 × 16 mm	8	0.146	1.168	steel
Hexagon socket head cap screws	M2 × 16 mm	1	0.063	0.063	steel
Hexagon socket head cap screws	M4 × 12	14	0.118	1.652	steel
Hexagon socket head cap screws	M4 × 20	3	0.175	0.525	steel
Hexagon socket head cap screws	M4 × 45	5	0.35	1.75	steel
Hexagon socket head cap screws	M4 × 25	4	0.086	0.344	steel
Hexagon socket head cap screws	M4 × 10	8	0.56	4.48	steel
Hexagon socket head cap screws	M5 × 16 mm	8	0.099	0.792	steel
Hexagon socket head cap screws	M2 × 6 mm	12	0.028	0.33	steel
Hexagon socket head cap screws	M3 × 10 mm	2	0.029	0.058	steel
Hexagon socket head cap screws	M3 × 16 mm	2	0.039	0.078	steel
Hexagon socket head cap screws	M3 × 25 mm	2	0.059	0.118	steel
Hexagon socket head cap screws	M3 × 40 mm	1	0.097	0.097	steel
Phillips screws	M2.9 × 16 mm	4	0.055	0.22	steel
Phillips screws	M2.2 × 4.5 mm	1	0.025	0.025	steel
Hexagonal nuts	M2.5	1	0.13	0.13	steel
Hexagonal nuts	M3	7	0.13	0.91	steel
Hexagonal nuts	M4	38	0.14	4.94	steel
Hexagonal nuts	M5	4	0.135	0.54	steel
Total			23.42		

Regarding costs, the aluminum alloy material used to fabricate the mobile chassis in Table 2 costs 181 CNY. The 3D-printed parts in Table 3 are produced using PLA with 30 % infill (79 CNY per kilogram), requiring 296.49 g and 659 min of printing time, at a cost of 24.43 CNY. The total cost of the electronic components in Table 4 amounts to 1756.26 CNY, while the mechanical components in Table 5 cost 23.42 CNY. Considering variations in material costs and market prices, the total cost of a single inspection robot is approximately 1985 CNY, excluding transportation fees for the components.

## 6 Build instructions

Once the preparation of the materials is complete, assembly of the physical prototype may begin. The assembly process should follow the steps outlined below and requires basic tools, including screwdrivers, wire strippers, pliers, a 3D printer, and a soldering iron.

### 6.1 Conventions and tips

To optimize usability during the assembly process, the following considerations should be noted prior to starting the structural assembly:

• Prior to assembly, verify and prepare all required components to ensure they are intact, free from damage, and confirm that all electronic components are functioning properly.
• Adhere to the step-by-step assembly sequence provided in this manual to minimize potential errors.
• Although rare exceptions may exist, the illustrations are generally organized according to the assembly sequence of the components and should be used as the primary reference.
• Screws and other threaded parts should not be fully tightened during the initial assembly phase.
• This manual provides diagrams for each step, illustrating the required components, their installation methods, and the positions of the assemblies, along with reference images of the final assembled state.
• The design of the robotic arm components is provided for reference only. To simplify manufacturing or optimize appearance, the design may be adjusted based on straight-line dimensions.
• The module models and parameters in this project may differ from those of the modules you currently possess. Adjustments should be made based on the documentation of the equipment in use.
• For safety reasons, do not connect the battery or any other power source during the assembly process.
• For ease of assembly, identical components in the illustrations are labeled with the same numbers throughout the manual.

**Fig. 10:**
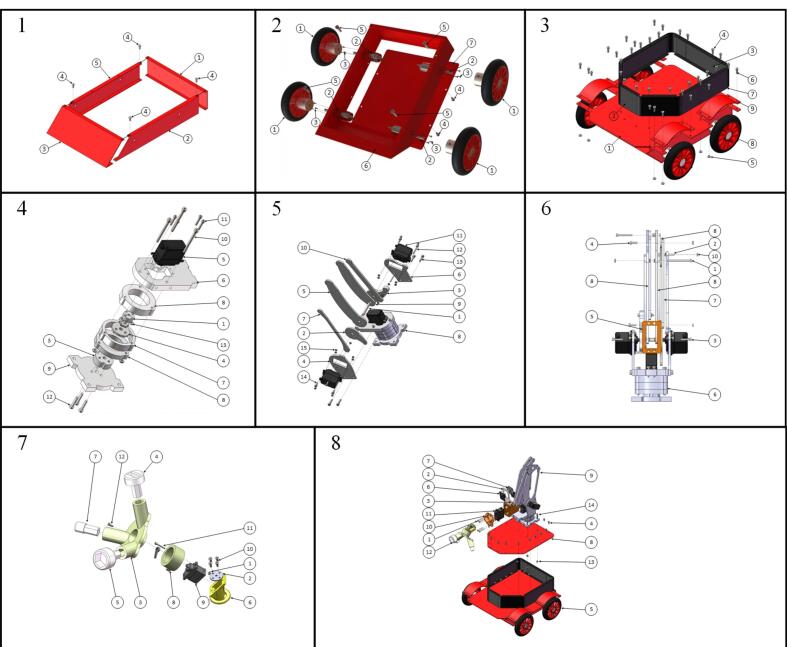
Assembly instructions.

The instructions for rapid assembly are illustrated in Fig. 10, and the detailed assembly procedures are described below.

### 6.2 Mobile chassis underframe

**Fig. 11:**
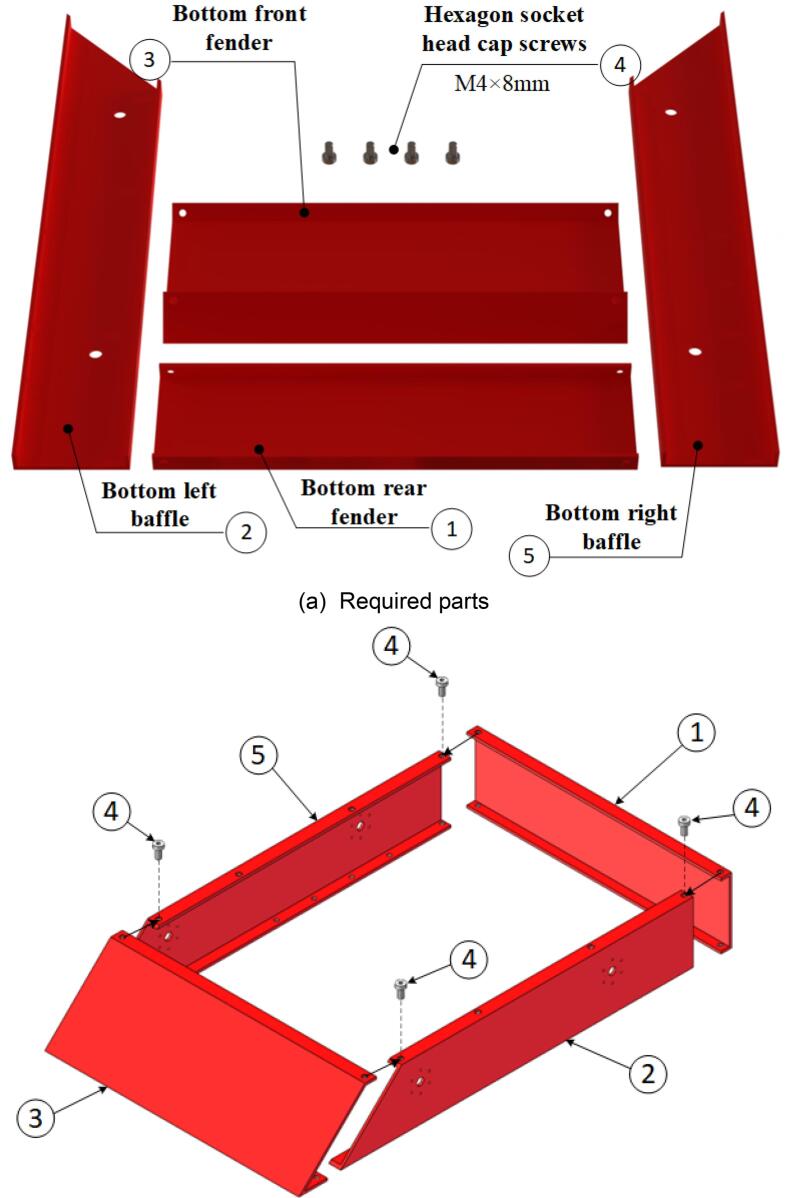

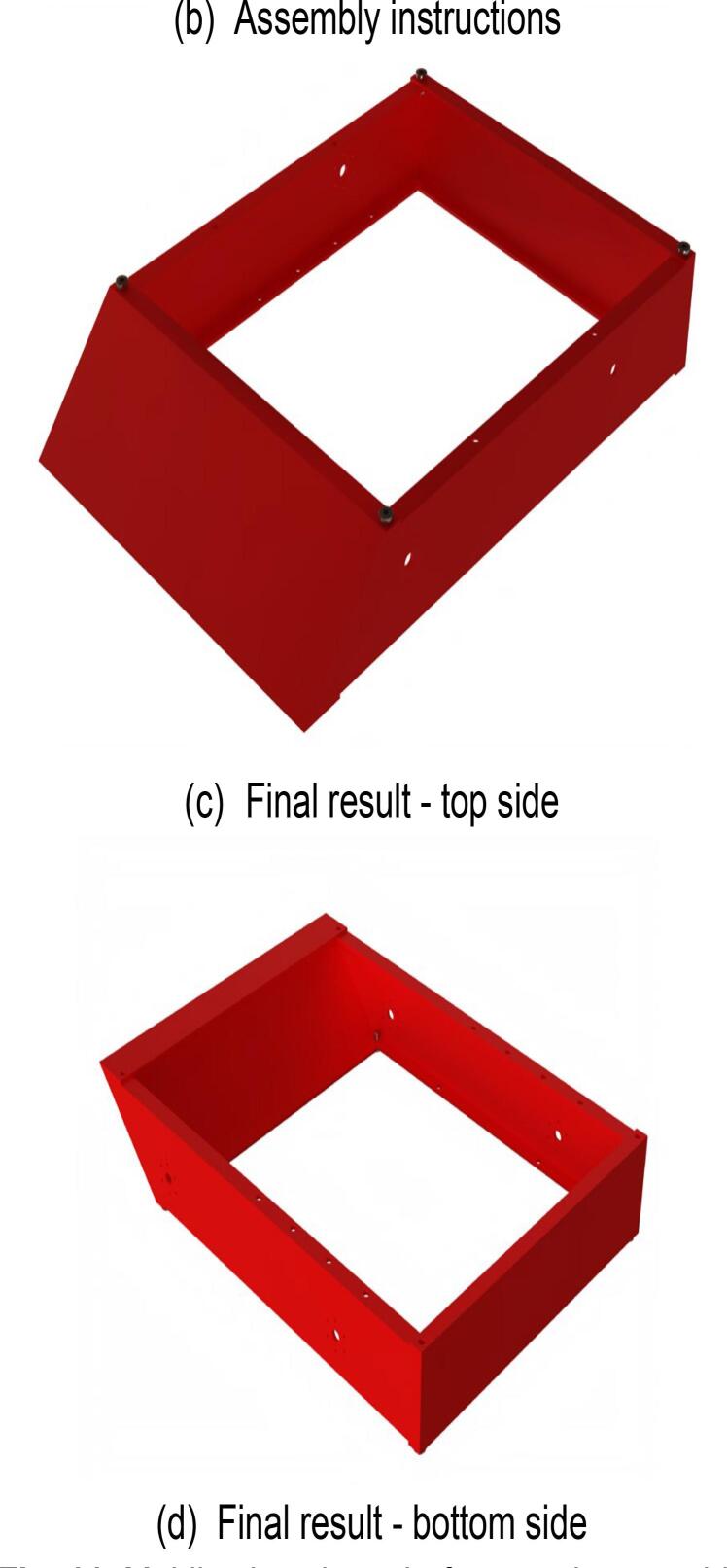
Mobile chassis underframe sub-assembly.

The steps for assembling the lower frame of the mobile chassis are shown in Fig. 11, with the required components listed in Fig. 11(a). During assembly, note that all screw-insertion points are equipped with threaded holes. Use a hex key wrench to tighten the screws into the corresponding components. The fully assembled lower frame of the mobile chassis is presented in Fig. 11(c) and (d).

### 6.3 Moving the lower part of the chassis

**Fig. 12:**
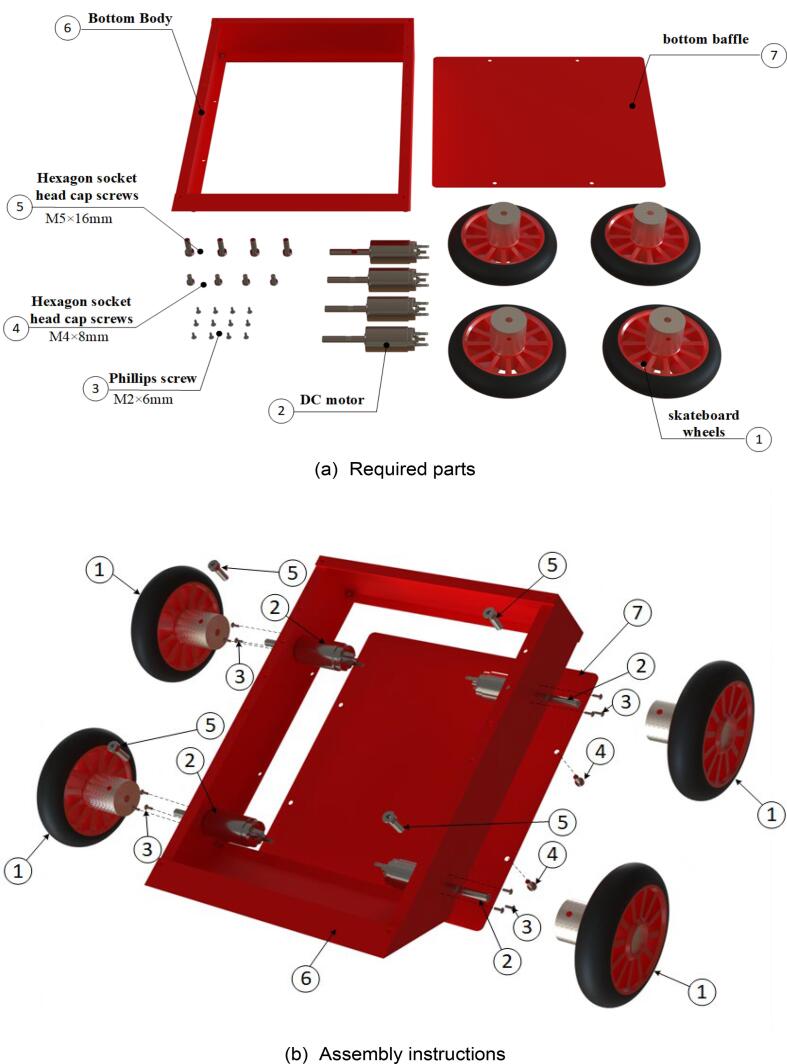

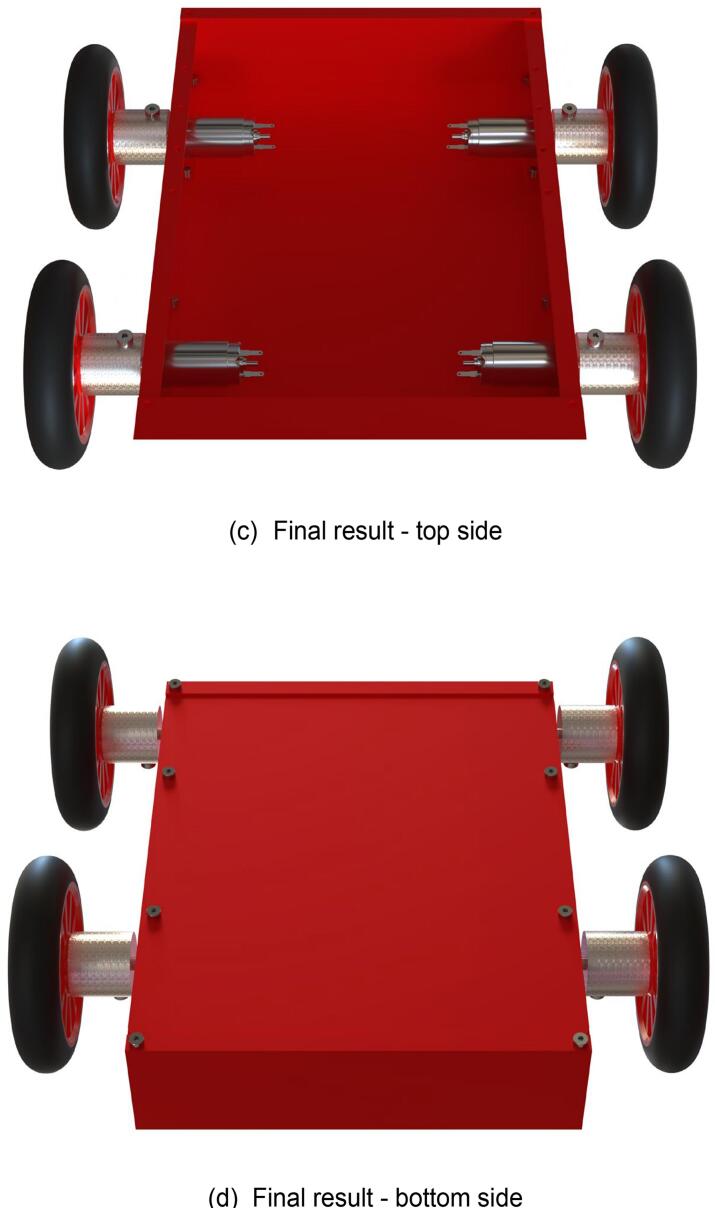
Moving the lower molecular assembly of the chassis.

The steps for assembling the lower section of the mobile chassis are illustrated in Fig. 12, with the required components listed in Fig. 12(a). During assembly, it is important to note that threaded holes are provided at all screw-insertion points. A hex key wrench should be used to tighten the screws and connect the components. As motor models may vary, the pre-drilled holes for motor installation may be adjusted based on requirements. The fully assembled lower section of the mobile chassis is shown in Fig. 12(c) and (d).

### 6.4 Mobile chassis

**Fig. 13:**
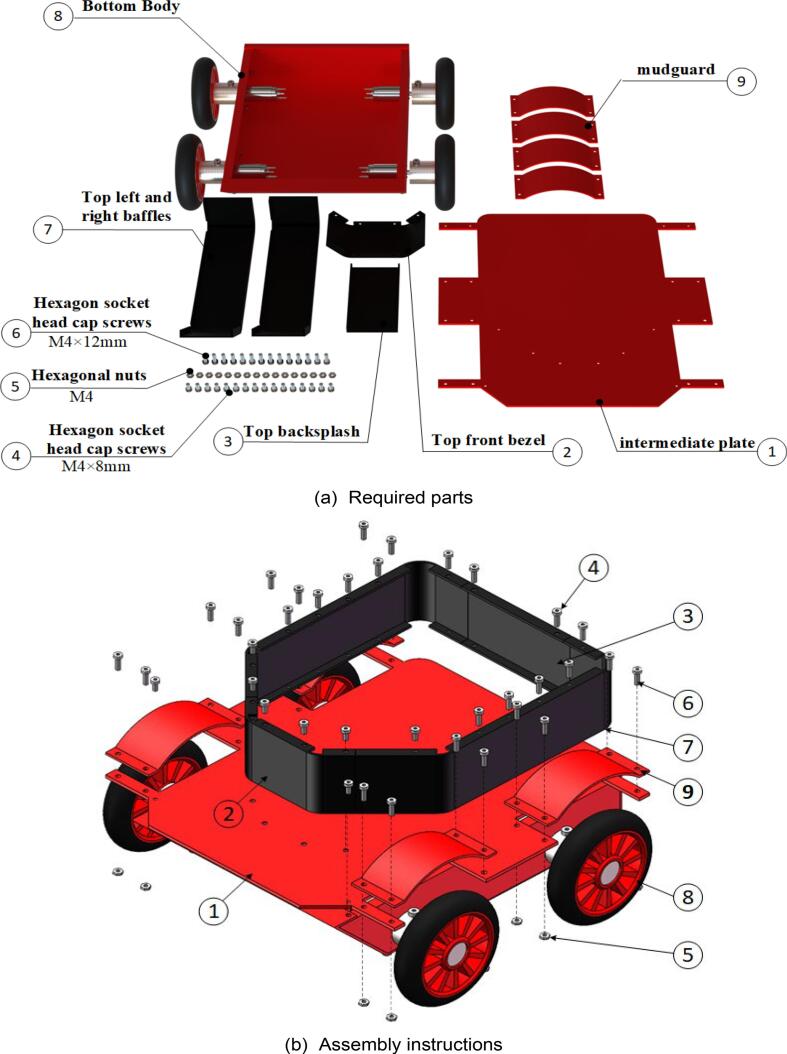

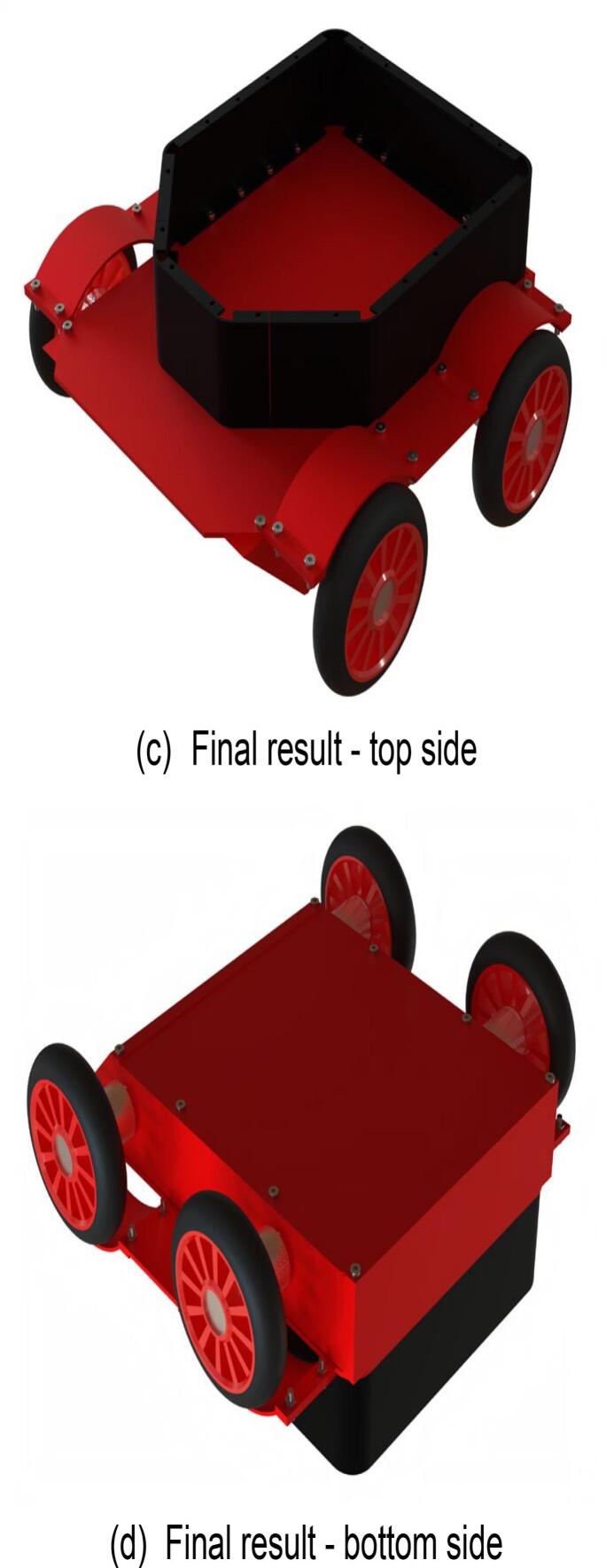
Mobile chassis sub-assembly.

The final stage of mobile chassis assembly involves completing the last step. The specific assembly steps are illustrated in Fig. 13, with the required components listed in Fig. 13(a). During assembly, note that threaded holes have been provided at all screw-insertion points. Use a hex key wrench to tighten the screws into the components. Special attention is required, as two types of screws are used in this step; ensure that each screw is installed in accordance with its specifications. The fully assembled mobile chassis is shown in Fig. 13(c) and (d).

### 6.5 Robotic arm base

**Fig. 14:**
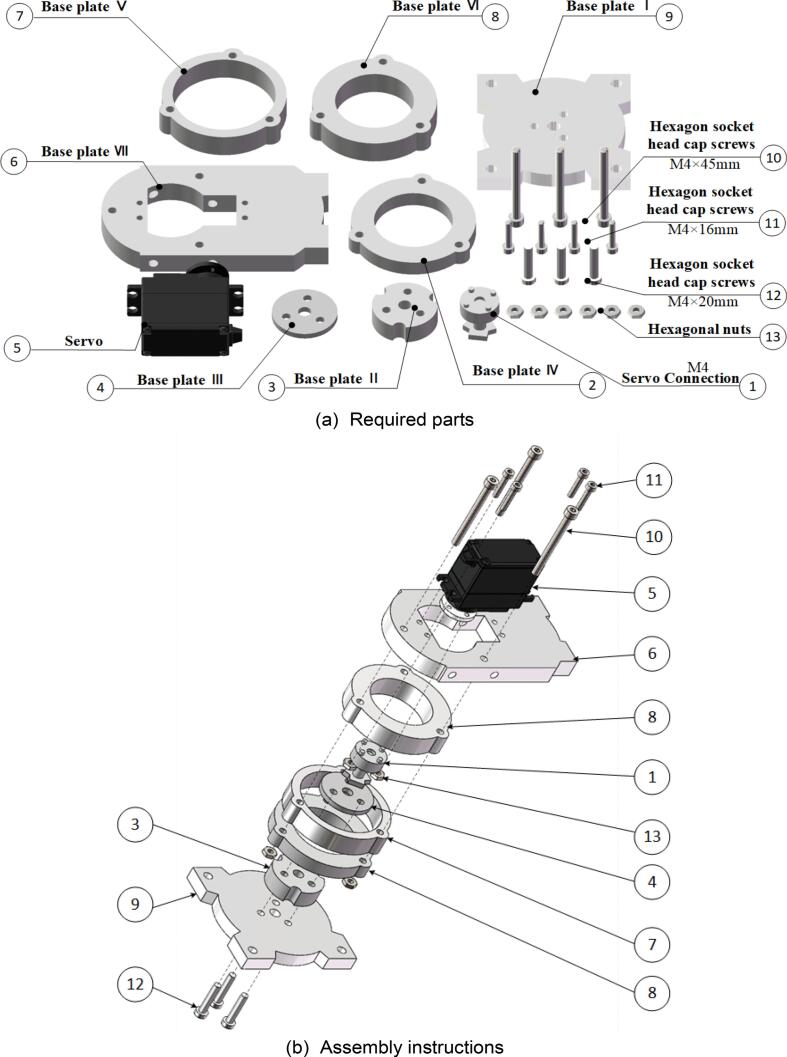

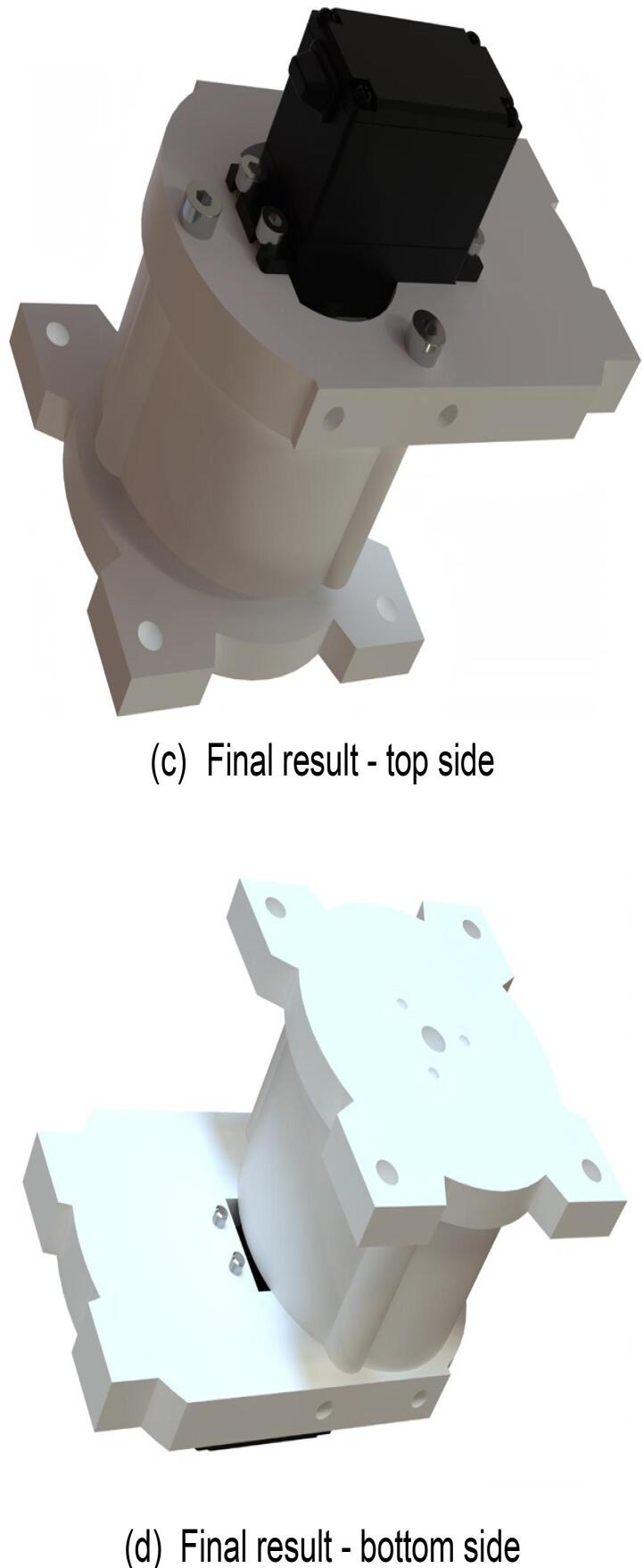
Robotic arm base sub-assembly.

Start the assembly of the robotic arm base. The assembly steps are illustrated in Fig. 14, and the required components are listed in Fig. 14(a). Ensure that each component is accurately installed to prevent misalignment or alterations. Use a hex key wrench to fasten the screws to the corresponding components. The fully assembled robotic arm base is shown in Fig. 14(c) and (d).

### 6.6 Robotic arm large arm

**Fig. 15:**
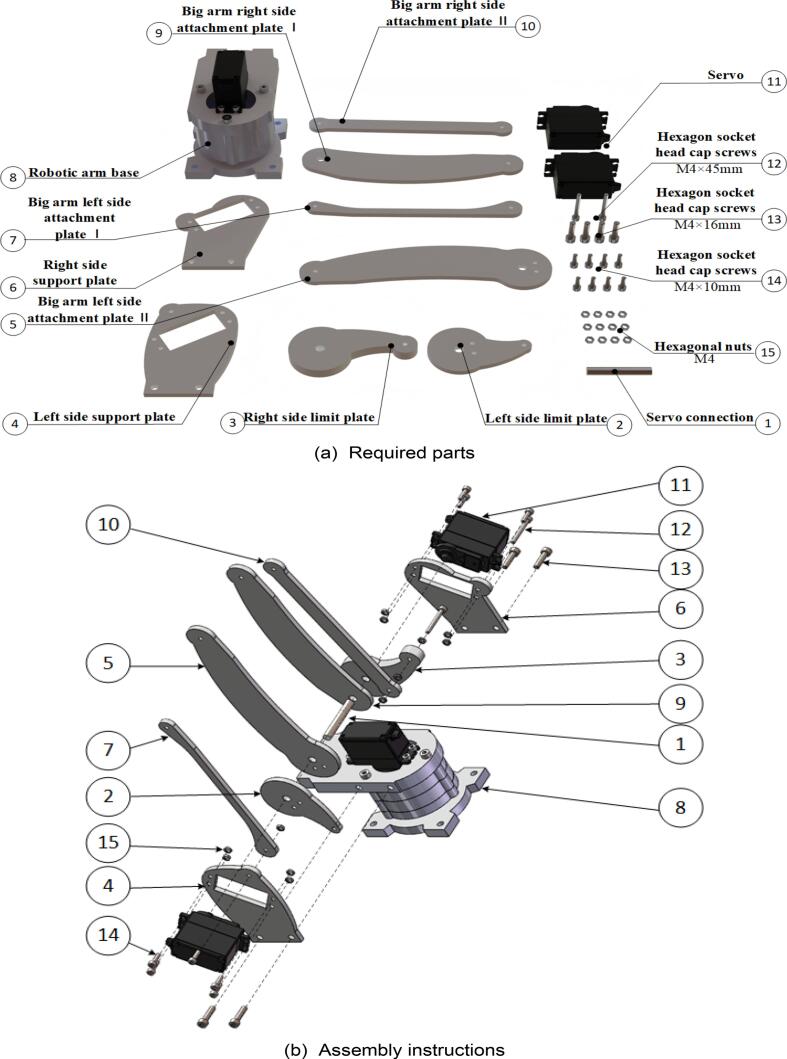

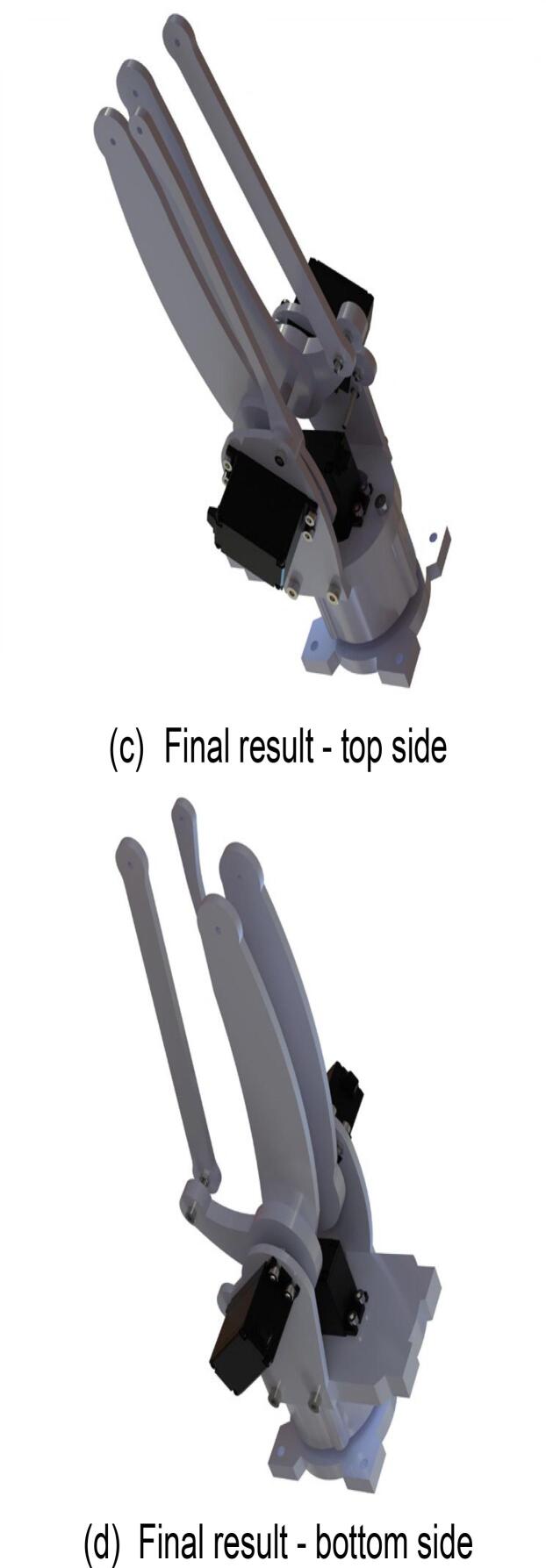
Robotic arm large arm sub-assembly.

Start assembling the robotic arm’s main arm. The specific assembly steps are illustrated in Fig. 15, and the required components are listed in Fig. 15(a). As some components of the main arm are highly similar, it is crucial to ensure proper identification and placement of each part. Use a hex key wrench to fasten the screws into the designated components. The fully assembled main arm of the robotic arm is shown in Fig. 15(c) and (d).

### 6.7 Robotic arm small arm

**Fig. 16:**
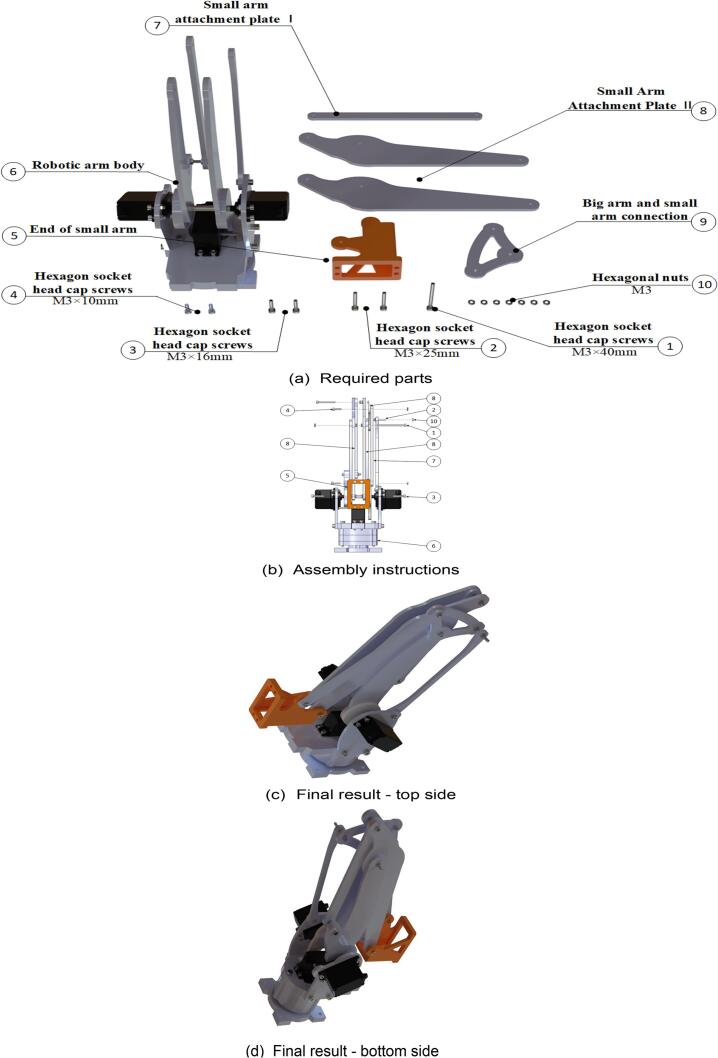
Robotic arm small arm sub-assembly.

Start the assembly of the robotic arm’s forearm. The specific assembly steps are illustrated in Fig. 16, and the required components are listed in Fig. 16(a). Use a hex key wrench to fasten the screws to the designated components. The fully assembled forearm of the robotic arm is shown in Fig. 16(c) and (d).

### 6.8 Manipulators

**Fig. 17:**
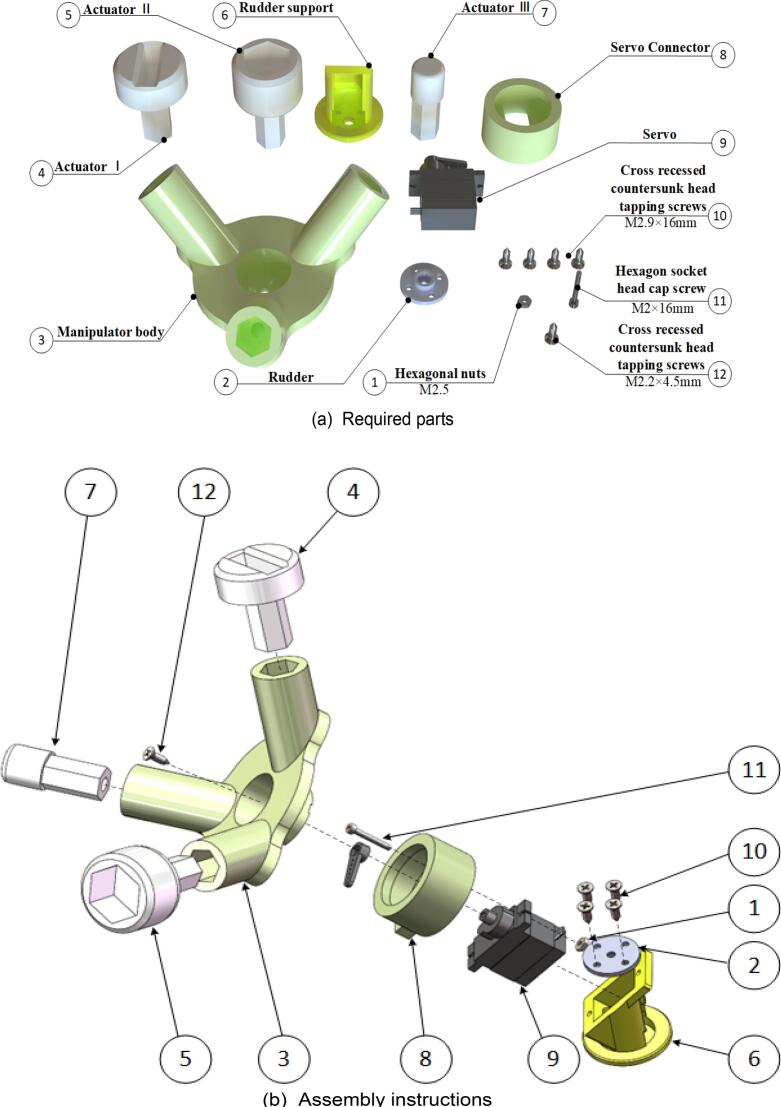

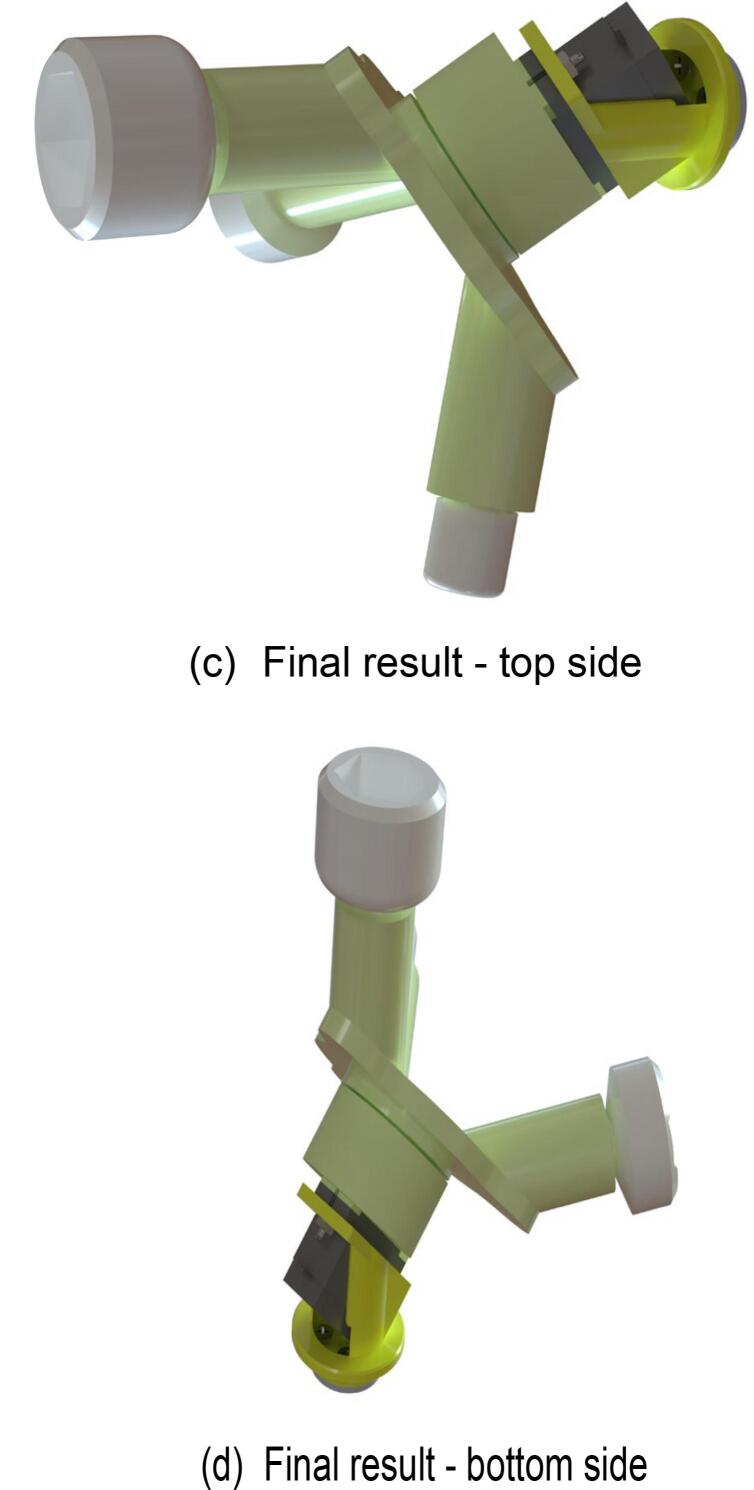
Robotic sub-assembly.

Begin assembling the robotic gripper. The specific assembly steps are illustrated in Fig. 17, and the required components are listed in Fig. 17(a). The gripper body is connected to the robotic fingers using magnets (not explicitly indicated in the assembly diagram). If adjustments are required, modify the dimensions of the gripper body and robotic finger models to match the size of the purchased magnets. Use a Phillips screwdriver to fasten the screws to the components. The fully assembled robotic gripper is shown in Fig. 17(c) and (d).

### 6.9 Mobile robot

**Fig. 18:**
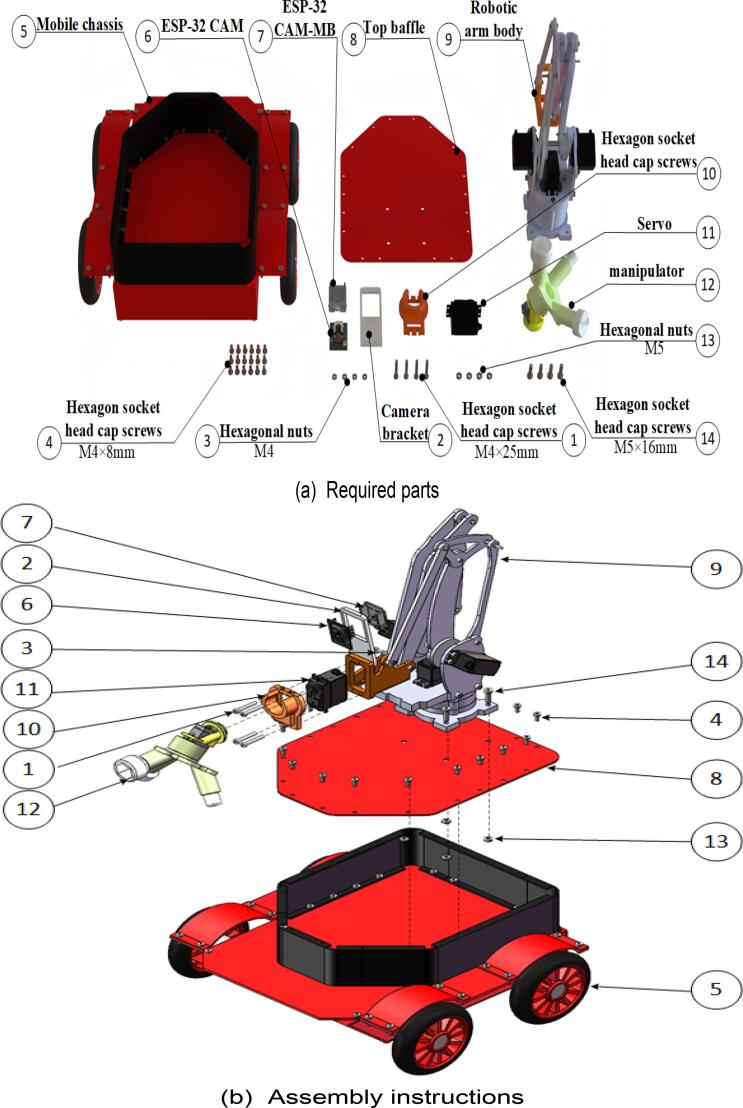

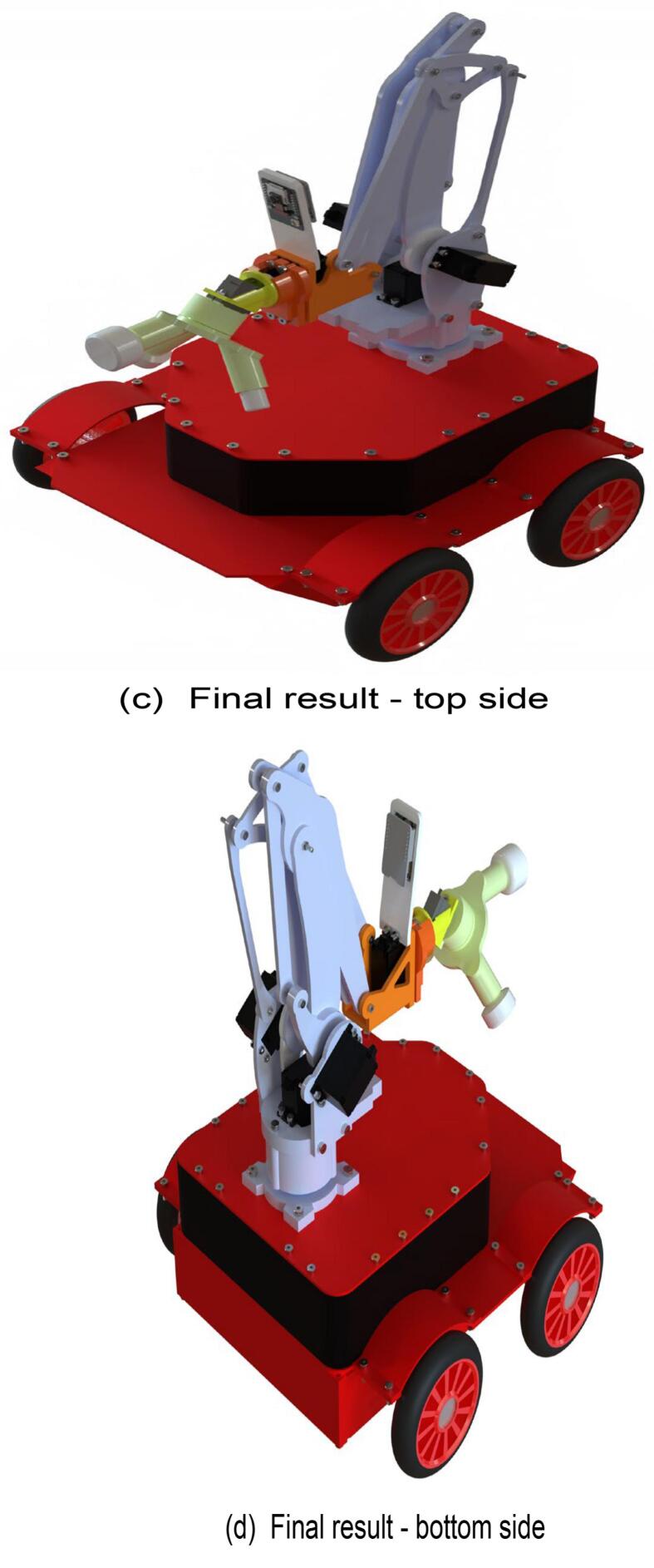
Mobile robot assembly.

Finally, proceed with the assembly of the mobile robot. The specific assembly steps are illustrated in Fig. 18, and the required components are listed in Fig. 18(a). Use a hex key wrench to secure the screws in the designated components. The fully assembled mobile robot is shown in Fig. 18(c) and (d).

### 6.10 Wiring and electronics

**Fig. 19:**
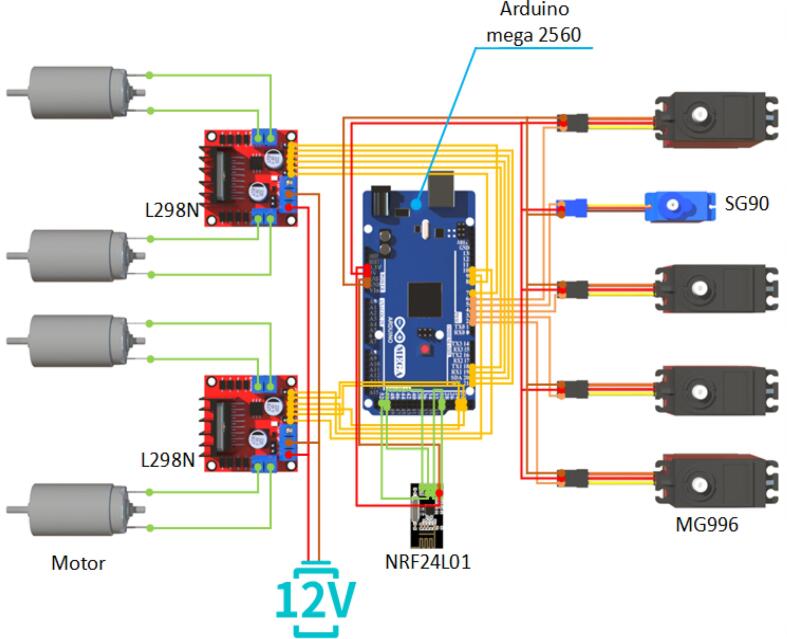
Wiring schematic for mobile robot side.

The wiring diagram for the mobile robot’s terminals is shown in Fig. 19. The Arduino MEGA 2560 is first connected to the V2 expansion board. The GND and VCC (3.3 V) pins of the NRF24L01 wireless transceiver module are connected to the GND and VCC pins on the V2 expansion board, respectively. The CE and CSN pins are connected to pins 30 and 31, respectively, as defined in the program. The MISO, MOSI, and SCK pins are connected to pins 50, 51, and 52, respectively.

The servo connections are as follows: the base servo is connected to pin 2, the upper arm servo to pin 3, the forearm servo to pin 4, the gripper rotation servo to pin 5, and the end-effector switching servo to pin 6.

The wiring between the L298N motor driver module, the motors, and the V2 expansion board is as follows: the output terminals of the L298N module are connected to the motor wires, while the positive terminal of the battery power supply is connected to the 12 V power input of the L298N module, and the negative terminal of the battery is connected to the GND of the power supply. The enable pins of the L298N module are connected to pins 7, 8, 9, and 10 on the V2 expansion board, while the logic input channel pins are connected to pins 18, 19, 20, 21, 22, 23, 24, and 25 on the V2 expansion board.

## 7 Operation instructions

### 7.1 Programming and Uploading

**Fig. 20:**
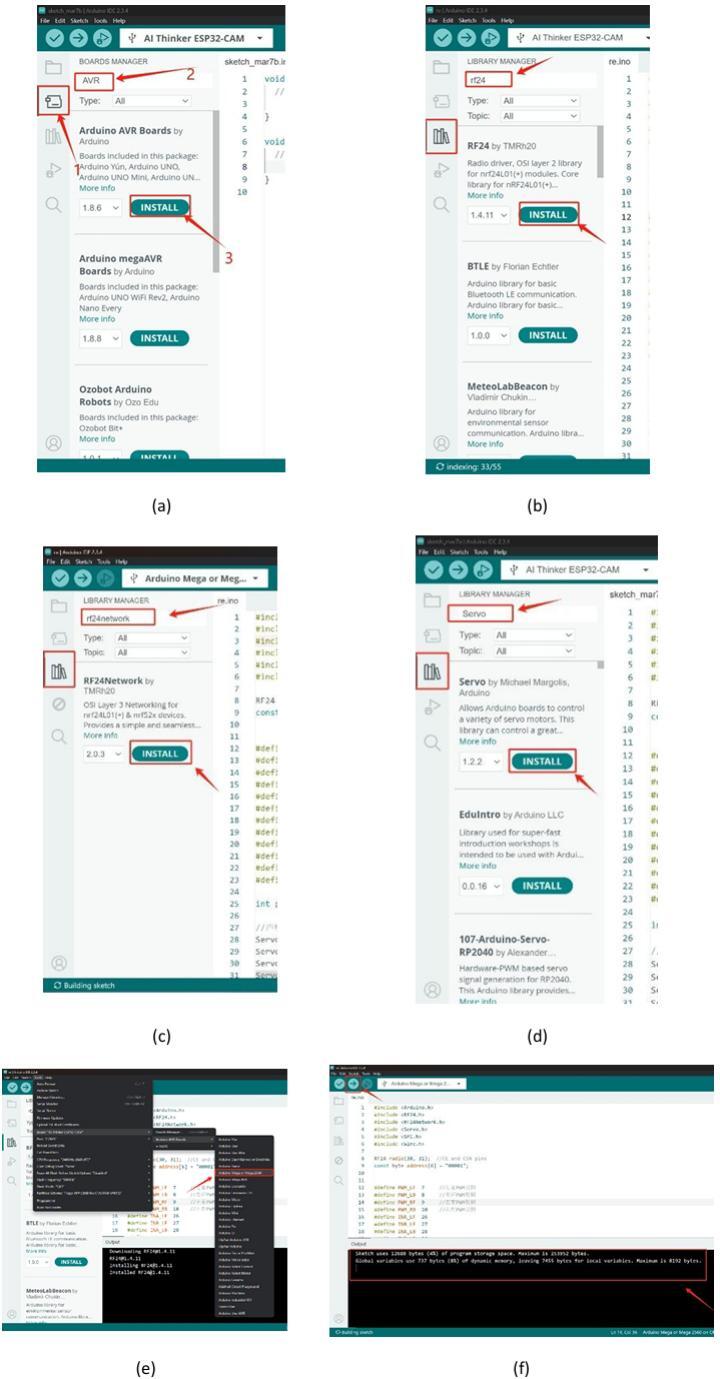
Firmware programming process.

Ensure that the development board is connected to the computer using a data cable, and open the Arduino IDE to configure the development environment. As shown in Fig. 20(a), click the arrow-indicated location, enter “AVR” in the search box, and install the corresponding result. Paste the main code from the provided receiver folder into the program editing window.

Proceed with the installation of the following library dependencies sequentially:

1. RF24 Library: As shown in Fig. 20(b), click the arrow-indicated location, search for “RF24″ in the search box, locate the result, and install it.
2. RF24Network Library: As shown in Fig. 20(c), search for “RF24Network” in the search box, locate the result, and install it.
3. Servo Library: As shown in Fig. 20(d), search for “Servo” in the search box, locate the result, and install it.

After completing the dependency installation, select the development board model as indicated in Fig. 20(e). Finally, upload the program to the board through the burning process, and verify the result as shown in Fig. 20(f).

### 7.2 Operating instructions for the inspection robot

**Fig. 21:**
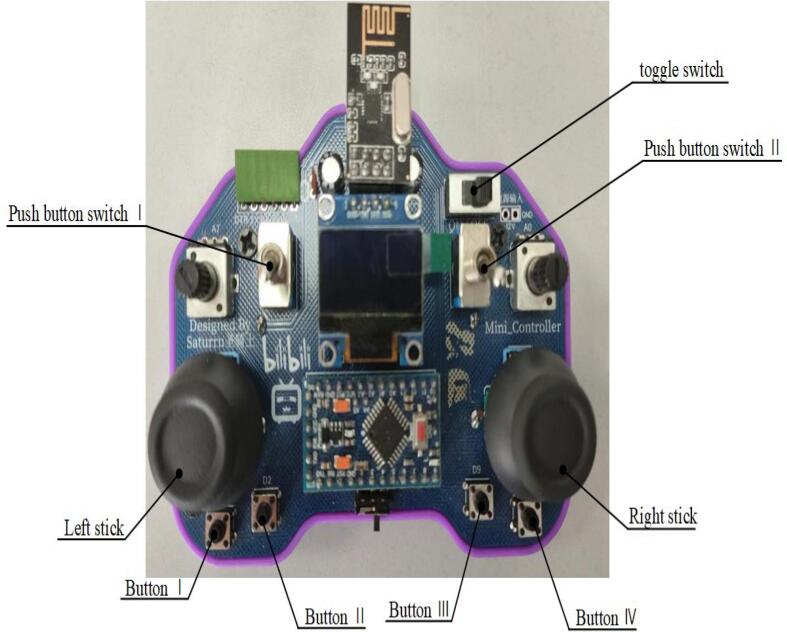
Remote control schematic.

The remote controller is shown in Fig. 21. First, turn on the power of the inspection robot, followed by the power of the remote controller. Once the robot is powered on, the blue light on the power button will illuminate, indicating that the system is ready for operation. The robot’s movement can then be controlled using the remote controller. The robot’s movement is controlled by the joysticks on the remote controller: Moving the joysticks upward, downward, left, or right controls the mobility of the mobile robot (chassis). The left joystick is specifically used for forward, backward, left, and right movement.

When the left joystick is moved downward, control of the robotic arm is activated: Moving the left joystick vertically controls the robotic arm’s X-axis movement at the end-effector. Moving the left joystick horizontally controls the Z-axis movement at the end-effector. The end-effector is operated using predefined functional buttons: Pressing buttons 2, 3, and 4 moves the end-effector to positions 1, 2, and 3, respectively. Additionally, pressing the left joystick activates the forward rotation of the end-effector, while pressing the right joystick activates reverse rotation.

## 8 Validation and characterization

### 8.1 Experimental simulation of the substation inspection robot

**Fig. 22:**
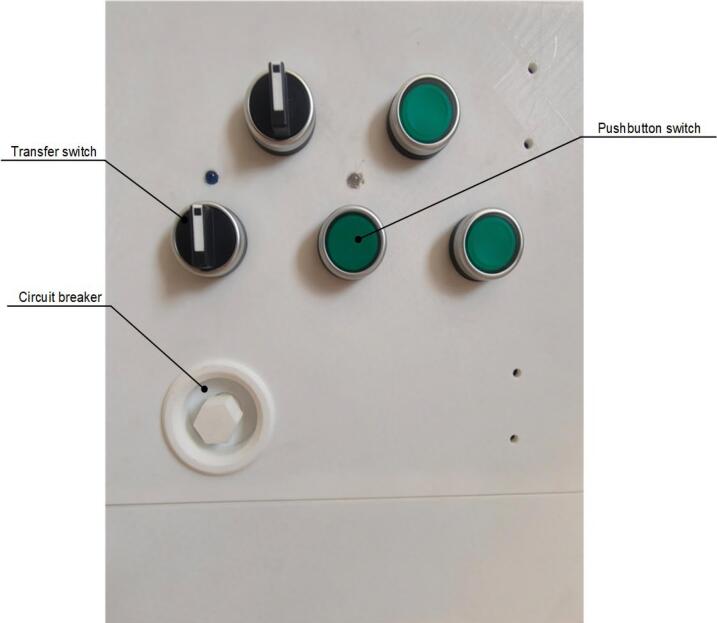
Switch position schematic.

A scaled-down model was constructed to scale with the dimensions of a power distribution cabinet. The model employs a wooden box-like structure to simulate the power distribution box, while the operation panel of the cabinet was fabricated using 3D printing. The panel includes buttons, rotary switches, and circuit breakers, with the switch positions shown in Fig. 22. The experimental environment is maintained at a room temperature of 20 °C, and the floor is made of wood. The test site must be equipped with a wireless network to ensure stable reception and real-time processing of transmitted images on the PC end. Additionally, the site should be shielded from intense lighting to ensure that operators can clearly observe the real-time images.

### 8.2 System validation experiment

To verify the functionality and performance of the inspection robot across a range of operational scenarios, a series of experiments was conducted. These experiments included operations involving button switches, rotary switches, and withdrawable circuit breakers, as well as adaptability testing under low-light conditions.

#### 8.2.1 End-effector button switch operation test

**Fig. 23:**
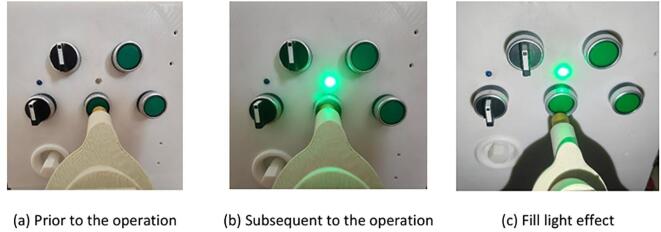
Button switch operation experiment.

The experiment simulated the end-effector’s ability to operate button switches. The operator remotely controlled the robot to position it in front of the power distribution cabinet, while real-time images were transmitted to the PC via the ESP32-CAM module. Based on the images displayed on the PC, the operator remotely controlled the end-effector to adjust its fingers and press the button switch. The experimental results demonstrated that the end-effector could accurately perform the button-pressing operation, successfully restoring the indicator light to its normal state. This verified the end-effector’s capability to operate button switches. Additionally, the experiment highlighted the effectiveness of the illumination enhancement under low-light conditions. The specific operational performance is shown in Fig. 23.

#### 8.2.2 End-effector rotary switch operation test

**Fig. 24:**
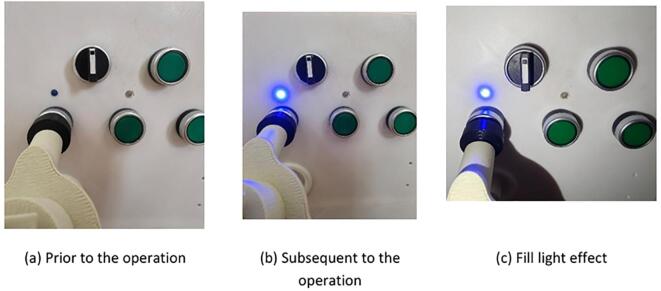
Knob switch operation experiment.

The experiment simulated the end-effector’s ability to perform rotational operations on rotary switches. The operator remotely controlled the robot to position it in front of the power distribution cabinet, with real-time images transmitted to the PC. Based on the images displayed on the PC, the operator remotely controlled the end-effector to adjust its fingers and rotate the rotary switch. The experimental results demonstrated that the end-effector could successfully rotate the rotary switch, restoring the indicator light to its normal state. Additionally, the experiment highlighted the effectiveness of the illumination enhancement under low-light conditions. The specific operational performance is shown in Fig. 24.

#### 8.2.3 End-effector circuit breaker operation test

**Fig. 25:**
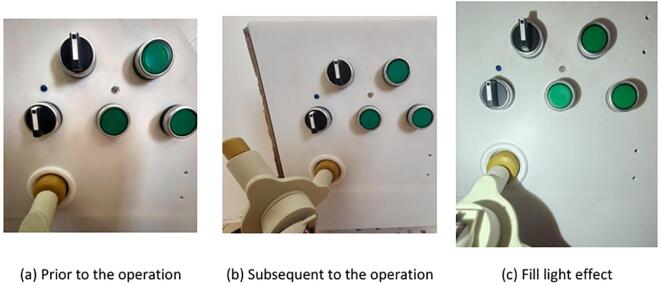
Handcart circuit breaker operation experiment.

The experiment simulated the end-effector’s ability to operate a circuit breaker. The operator remotely controlled the robot to move it into position in front of the power distribution cabinet, while real-time images were transmitted to the PC via the ESP32-CAM module. Using the images displayed on the PC, the operator remotely controlled the end-effector to adjust its fingers and operate the circuit breaker. The experimental results demonstrated that the end-effector could accurately operate the circuit breaker. Additionally, the experiment highlighted the effectiveness of the illumination enhancement under low-light conditions. The specific operational performance is shown in Fig. 25.

## 9 Conclusion

This paper proposes a modular manipulator-based inspection robot for power distribution substations, designed to address the limitations of traditional inspection robots, such as slow tool switching and insufficient operational flexibility. By adopting a coaxial and collinear design for the end-effector, the manipulator achieves rapid switching between three tool positions with single-degree-of-freedom actuation, enabling precise execution of various tasks, including button switches, rotary switches, and draw-out circuit breakers. Experimental validation demonstrates that the stress distribution of the modular manipulator in various operational scenarios meets the design strength requirements, ensuring its reliability and durability. Additionally, the robot adheres to low-cost, open-source, and modular design principles, reducing manufacturing and maintenance costs while providing a flexible hardware platform for experimental research. The innovation of this study lies in the design of the modular manipulator and its application in substation inspection, providing a novel technical pathway for future unmanned substation inspections.

However, there are some limitations in this study. First, the environmental adaptability of the inspection robot requires further improvement, including performance evaluation in complex terrains or under extreme conditions like high humidity and high temperatures. Second, the robot currently relies on manual remote operation. Although its functionality meets the basic inspection requirements, it still has significant room for improvement in achieving full autonomy. Future research will focus on optimizing hardware configurations, incorporating sensors such as LiDAR and stereo cameras, and implementing the Robot Operating System (ROS) to enhance the automation and intelligence of the inspection robot, thereby supporting the intelligent operation and maintenance of power systems.

## Declaration of competing interest

The authors declare that they have no known competing financial interests or personal relationships that could have appeared to influence the work reported in this paper.
